# Humanitarian data infrastructures for missing migrants: A multimodal and ethics-integrated framework

**DOI:** 10.12688/openreseurope.23124.1

**Published:** 2026-04-16

**Authors:** M. Fevzi Esen, Ecenur Aydemir

**Affiliations:** 1 Department of Health Information Systems, Hamidiye Institute of Medical Sciences, University of Health Sciences, Istanbul, Turkey; 2 Department of Healthcare Management, Faculty of Health Sciences, Acibadem Mehmet Ali Aydinlar University, Istanbul, Turkey

**Keywords:** Missing migrants; migration governance; sociotechnical systems; digital traces; humanitarian informatics; early warning systems.

## Abstract

The disappearance of migrants is a global humanitarian crisis aggravated by structural data deficiencies across fragmented migration routes. This study adopts a sociotechnical systems perspective to develop an ethics-integrated infrastructure designed to convert multimodal digital traces into actionable intelligence for search, identification, and policy accountability. We propose a novel typology classifying 32 trace types across ten domains ranging from geospatial markers to psychosocial signals like emoji usage, facilitating the systematic analysis of decentralized digital evidence. This classification underpins a modular architecture comprising five interconnected cores: ingestion, preprocessing, evidence integration, AI, and analysis. The system enables entity resolution and spatiotemporal reconstruction while incorporating provenance tracking and explainable AI to deliver early warnings and OCHA-aligned decision support. By embedding stringent cross-layer safeguards including dynamic consent, auditability, and interoperability, the framework ensures adherence to FAIR data principles and international human rights standards. This research offers a scalable, replicable method for validating digital evidence across the migration cycle, providing a bridge between computational innovation and rights-based migration governance. Future work targets operational pilots to assess feasibility and impact on triage efficiency, identification accuracy, and family notification protocols.

## 1. Introduction

The disappearance of migrants is an urgent global concern. According to Missing Migrants Project, the International Organization for Migration (IOM) has documented more than 75,000 missing persons along migration routes since 2014 ( IOM, 2025). Traditional systems like border registers and forensic databases often fail to capture the full scope of these incidents due to fragmented surveillance, reporting delays, and geopolitical constraints ( Obinna, 2025). With the expansion of digital connectedness, migrants and their communities are progressively producing real-time, socially embedded traces such as geotags, multilingual appeals, and visual evidence on platforms like X, Facebook, and TikTok ( Wilkinson and Castaneyra-Ruiz, 2021). Despite their potential, these traces are underutilized because of unstructured formats, ethical concerns, and institutional resistance ( Wahid et al., 2025). Consequently, families face persistent underreporting, knowledge gaps, and prolonged uncertainty.

Digital platforms generate diverse traces, including posts, hashtags, geotags, and interaction patterns. These provide time-sensitive, socially embedded signals that may enhance official sources. Translating heterogeneous, multilingual, and emotionally charged data into operational intelligence is difficult without typologies, interoperable workflows, and ethics-integrated governance. Previous research has explored social media analytics in the contexts of disaster response and refugees; however, a comprehensive framework specifically addressing irregular migrant disappearances is lacking. Existing studies focus on isolated data types, neglecting the integration of multimodal traces and ethical governance ( Leasure et al., 2023). This gap limits the development of field-ready systems that uphold dignity, privacy, and accountability.

To address these multifaceted challenges, this study adopts a Sociotechnical Systems (STS) perspective, conceptualizing humanitarian data infrastructures as ensembles where technical artifacts, institutional policies, and human actors intersect. By viewing digital traces not merely as neutral inputs but as socially situated signals, this framework addresses the entire “migration cycle” from the initial distress in transit to the legal identification of missing persons. This approach ensures that technical innovations, such as the proposed modular architecture, are fundamentally grounded in Data Justice, facilitating a rights based response that supports both governmental policy objectives and the lived experiences of affected communities.

To bridge the fragmented surveillance and ethical governance gaps identified above, this paper provides a three-fold conceptual contribution. First, it establishes a comprehensive typology that categorizes 32 distinct digital trace types across 10 domains, spanning geospatial markers to psychosocial signals. Second, it introduces a modular, ethics-integrated framework for humanitarian data infrastructures designed to govern the responsible collection and processing of these traces. Finally, it demonstrates how principles of data stewardship, privacy, and accountability can be practically embedded into interoperable information systems ready for field deployment.

This study is organized according to the following research questions, aligned with these objectives:

1. How can social media platforms be routinely utilized to collect and analyze data pertinent to missing migrants?
2. What kind of operational value can be obtained from content disseminated by families and communities?
3. Which technical instruments and analytical techniques, such as artificial intelligence and multimodal data analysis, are most efficacious for processing and integrating social media content in this context?
4. What ethical and privacy concerns must be considered while managing sensitive, user-generated data related to missing migrants?
5. How can insights derived from social media analysis be converted into meaningful initiatives for governments, NGOs, and humanitarian organizations?

The article is structured to address above mentioned questions systematically. Section 2 examines the constraints of existing humanitarian and migratory data infrastructures while exploring the potential of digital traces as supplementary evidence. Section 3 establishes the theoretical grounding in sociotechnical systems and data justice. Section 4 presents the proposed typology and modular architecture in detail, illustrating their role in facilitating ethically regulated identification processes. Finally, the paper delineates the governance challenges and policy implications essential for the secure integration of these technologies into global humanitarian practice.

## 2. Background

Humanitarian responses to missing migrants have traditionally relied on institutional data systems, including border control records, missing persons registries, forensic archives, and international databases. Nevertheless, extensive study highlights persistent structural limitations:

• Spatiotemporal fragmentation: Notable coverage deficiencies arise in isolated, high-risk areas, including coastal pathways and desert traverses, leading to underreporting and "informational blind spots" ( UNSD, 2025).
• Procedural delays: Bureaucratic processes, inconsistent reporting, and geopolitical constraints impede the prompt exchange of information ( Calderón et al., 2024; Tefera and Gamlen, 2025).
• Identification failures: Insufficient physical evidence, delayed notifications, and constrained access to official systems result in numerous missing persons remaining unidentifiable. Even prominent projects, such as the Missing Migrants Project, encounter difficulties due to their dependence on disjointed and retrospective statistics from NGOs and official entities ( Black, 2024). These limitations underscore the need for real-time supplementary data.

### Digital traces as complementary humanitarian sensors

Research in migration studies and digital ethnography indicates that social media enables decentralized solutions to crises ( Rejali and Heiniger, 2020; Leurs & Ponzanesi, 2024; Sithole, 2024). Unlike conventional infrastructures, digital platforms enable near-real-time data acquisition, decentralized validation, and rapid support network mobilization ( Erokhin and Komendantova, 2024; Herrera et al., 2025). Methods like crowdsourcing in emergencies and extensive digital humanitarianism illustrate how community-oriented platforms can produce actionable intelligence ( Dave, 2017; Poblet et al., 2018).

Migrants use social media to seek help, plan routes, and stay connected ( Talabi et al., 2022). This activity generates both intentional posts and contextual "ambient" traces such as geotags, network topologies, and cross-platform interaction patterns that indirectly indicate mobility, community building, and vulnerability ( Walk et al., 2023). Social media, as a responsive “social sensor”, helps detect migration distress signals ( Theja Bhavaraju et al., 2019).

Reviews show that mobile phone metadata and internet searches can estimate migration flows, intentions, and integration ( Tjaden, 2021). For instance, studies on Syrian asylum seekers show mobile phones are essential for navigation but also expose users to risks like surveillance and misinformation ( Dekker et al., 2018).

### Analytical frameworks for heterogeneous data

Analyzing diverse digital traces requires frameworks that unify multiple data types into actionable insights. Artificial Intelligence (AI) techniques, especially Natural Language Processing (NLP) and Deep Learning (DL) are pivotal in categorizing crisis-related content, performing sentiment analysis, and discerning user roles ( Rabani et al., 2023). Topic modeling tools also facilitate the identification of emerging themes in extensive text corpora, whilst spatio-temporal analysis methods assist in route mapping, needs grouping, and the delineation of effect zones ( Kankanamge et al., 2020; Villodre and Criado, 2020).

Recent advancements, including the Joint Spatio-Temporal Topic-Sentiment framework and Topic2Labels provide scalable, multimodal methodologies that diminish dependence on manually annotated datasets; however, their efficacy is contingent upon the adaptation of models to the linguistic and cultural contexts of migration ( Hanny and Resch, 2025; Deng et al., 2020).

### Governance, ethics, and responsible implementation

Despite increases in analytical capability, structural constraints remain to prevent the ethical and operational integration of digital trace analytics in missing migrant studies. These challenges cover four interrelated domains:

1. Ethical problems (e.g., algorithmic bias, surveillance exposure);
2. Technical limits (e.g., deficiencies in multilingual NLP capabilities, interoperability difficulties);
3. Governance deficiencies (e.g., absence of consent protocols, institutional opposition);
4. Methodological deficiencies (e.g., excessive dependence on text-based data, disregard for ambient traces).

These issues worsen migrant vulnerabilities and erode trust and data utility. Table 1 consolidates these multifaceted difficulties and presents the proposed framework as a cohesive solution.

**Table 1.** Multidimensional challenges and humanitarian implication.

Challenge domain	Key issues	References
Ethical risks	Surveillance exposure, algorithmic bias (e.g., Western-centric NLP models), voyeurism, retraumatization	Leasure et al., 2023; Spijkerboer, 2023; Jørgensen, 2024; OCHA, 2025; OHCHR, 2025; UNICRI, 2025
Technical limitations	Low-resource language support, interoperability gaps, misinformation proliferation	Gillespie et al., 2018; Santhanam et al., 2019; Rabani et al., 2023; Düvell, 2024
Institutional barriers	Restrictive data policies, bureaucratic resistance to crowdsourcing, legal ambiguities	De Hoon et al., 2019; Bélanger and Candiz, 2019; Mohamed et al., 2020; Haller and Yanaşmayan, 2023
Methodological gaps	Overreliance on single data types (e.g., Twitter text), neglect of ambient/emergent traces (e.g., AR filters, voice notes)	Kreutzer et al., 2025; Zhang et al., 2025; Zhou et al., 2025

The FAIR principles and comprehensive humanitarian ethics frameworks are still inadequately employed ( Wilkinson et al., 2016). Limited research integrates dignity-centered governance measures such as permission processes, bias audits, and provenance tracking across the whole data lifecycle ( Mohamed et al., 2020; Kreutzer et al., 2025). In response, this study aims to develop a comprehensive typology of digital data sources from geotags to ambient traces and map them within a humanitarian data infrastructure framework. The proposed framework addresses these gaps through embedded cross-layer controls, including algorithmic auditing and dynamic consent mechanisms. This also combines methodological innovation with ethics-integrated governance to improve timeliness, localization, and inclusivity in humanitarian action while maintaining dignity, privacy, and accountability.

## 3. Theoretical framework: Data justice and sociotechnical humanitarianism

The ethical and technical design of humanitarian data infrastructures requires more than functional innovation. It demands a critical lens on how such systems influence power relations, determine inclusion, and shape legitimacy in humanitarian contexts. This article builds its framework on three interrelated areas of scholarship: sociotechnical systems theory, critical data studies, and data justice in humanitarian technology. These theoretical perspectives shape the infrastructure's structure and ethical orientation, influencing how data is ingested, modeled, and governed. The pipeline design reflects these principles through its modularity, transparency, and integration of survivor-led mechanisms for validation and oversight.

### Sociotechnical systems in humanitarian contexts

Humanitarian technologies operate within institutions, cultures, and power structures that shape how data is produced, interpreted, and applied. The concept of sociotechnical systems draws attention to this interaction, highlighting that technologies are developed and function within the constraints and norms of their environments ( Kitchin, 2022). In crisis response, this means data is never separate from the social and political forces that surround it.

Digital traces shared through platforms like WhatsApp or Instagram illustrate this dual nature. While they provide potential insight into displacement events, they are also shaped by platform dynamics, linguistic choices, and network structures. Treating them as neutral inputs strips them of social meaning. In designing a humanitarian data infrastructure, responsiveness to these embedded social dynamics becomes necessary. Studies such as Taylor (2021) emphasize models that support distributed oversight and ethical flexibility. These priorities are reflected in the modular pipeline architecture described in this study.

### Critical data studies and the politics of traces

Traces are not passive records. They are constructed, selected, and sometimes ignored based on the priorities and assumptions of the systems that handle them. Critical data studies offers tools to analyze how this happens, especially for populations who fall outside formal systems of registration or legal recognition ( Iliadis & Russo, 2016).

Infrastructures that gather migrant data often function under conditions of uncertainty and fragmented access. Within this space, signals such as “last seen” timestamps or fragmented message histories may become central to locating someone, but their meaning is always shaped by context. Grohmann (2025) conceptualize data infrastructures as contested spaces where recognition, authority, and interpretation are negotiated. Hoffmann (2020) further critiques how systems that seek to include marginalized subjects may reproduce harm when the frameworks used to classify or validate information remain unexamined.

To address these dynamics, the framework includes mechanisms for trace provenance, consent pathways, and feedback loops. The system treats traces as ethically situated and context-dependent. They are not merely evidence to be verified or rejected, but socially meaningful inputs within a broader investigative process.

### Data justice and the ethics of humanitarian AI

Data justice provides a foundation for evaluating how humanitarian systems operate in practice. The concept shifts attention from technical performance or privacy to broader concerns around agency, representation, and accountability ( Taylor, 2017; Heeks & Renken, 2018). These concerns are particularly relevant where algorithmic systems are used to process sensitive or ambiguous information, such as signals relating to disappeared individuals.

Recent scholarship has emphasized the importance of making algorithmic governance both transparent and reflexive. Srivastava (2023) describe how decision-making under uncertainty, particularly in high-stakes contexts, demands systems that accommodate doubt and allow for external oversight. Within this framework, modularity supports this goal by enabling adaptation across institutional contexts while maintaining ethical consistency. The system’s architecture supports varied implementations by forensic experts, humanitarian organizations, or family-led groups, while keeping auditability and explainability intact.

Madianou (2019) stress that humanitarian innovation should be evaluated through the lens of justice rather than efficiency or scalability alone. In response, this framework incorporates participatory governance tools, enabling affected individuals and communities to engage in annotation, feedback, and redress. Ethical oversight is not relegated to post-hoc review but is embedded throughout the data lifecycle.

### Ethical traceability and system design

The typology of different trace types within this framework functions not only as a technical mapping, but also as an epistemological structure. Traces are historically and ethically situated. They are not isolated signals but elements within broader systems of meaning, risk, and recognition. These dynamics align with Milan and Treré’s (2020) analysis of how marginalized populations navigate datafication through resistance, improvisation, and situated knowledge production. Rather than pursuing maximal visibility or full data integration, the system prioritizes trace integrity, modular governance, and flexible implementation. Rights-based guidance, including OHCHR’s (2021) framework for data use in humanitarian settings, underpins the system’s ethical orientation. Each module in the pipeline, from data ingestion to AI modeling, is designed to support contextual reasoning, accountability, and trace-level review.

## 4. Results

### Typology of digital data sources

This section classifies digital data sources relevant to the inquiry of missing migrants, particularly from social and digital media. Table 2 classifies these sources into thematic categories that encompass certain data kinds. These categories reflect the complexity of digital traces in migration.

**Table 2.** Data sources in missing migrants research.

Category	Data Type	Use	Notes	Category	Data Type	Use	Notes
Content	Text	Posts, tweets, captions, comments	Core for NLP and content analysis	Media	Image and Video Content	Photos, embedded videos	Rich in direct visual information
	Multilingual Texts	Posts in Arabic, French, etc.	Migrant and diasporic communities		Reverse Image Search Metadata	Detects reused or manipulated images	Useful in misinformation detection
	Auto-Translated Texts	Posts using built-in translation	Useful for studying cross-lingual flows		Visual Recognition Tags	AI-detected objects, faces, scenery	Enables crowd, terrain, or facial recognition
	Pinned Posts	Prioritized or fixed user/page content	Signals urgency or awareness campaigns		Alt Text/Accessibility Labels	Textual metadata for images/videos	Useful for alternative or automated extraction
Interaction	Conversation Threads	Comment chains and replies	Used for discourse and misinformation tracking	Technical Metadata	Timestamps (UTC/Local)	Exact time of post	Reconstructs event timelines
	Live Streams-Real-Time Reactions	Interactive comments during live events	Often used during crisis or disappearance events		Platform or App Used	e.g., “Facebook for Android”	Indirect location/device proxy
Location	Locations/Check-ins	Geotags added to posts	Helps trace last-known location		Edit History	Tracked changes in posts	Flags narrative shifts or manipulations
	Inferred Locations via NLP	Place names mentioned in text	Extracted with geoparsing tools	External Signals	Shared URLs and Metadata	Outbound links to news/media	Media influence and source verification
	Spatiotemporal Mobility Patterns	Tracked over multiple posts by same user	Requires longitudinal data linking		Algorithmic Trends	What gets boosted, shadowed	Requires interpretive reverse engineering
Networks & Virality	Mention Networks	Who tags or mentions whom	Enables social graph or kinship tracing		Crowdsourced Alerts	Posts requesting public help (e.g., #WhereIsX)	Crucial for community search coordination
	Repost/Retweet Trees	Paths of content duplication	Used for rumor or misinformation spread tracking	Experimental&Emerging	Ambient Digital Traces	Last seen online, typing, read receipts	Used in presence detection (e.g., WhatsApp)
	Hashtag Co-occurrence	Clusters of hashtags	Thematic and activist network mapping		Voice and Audio Posts	Voice tweets, voicemails in DMs	Underused modality with emotional value
Semantic & NLP	Emotion & Sentiment Analysis	Expressions of anger, fear, hope, etc.	Affects community dynamics and policy response		GIFs and Memes	Encoded cultural and political expression	Rich in symbolism, humor, resistance
	Topic Modeling/Clustering	Unsupervised theme extraction	Methods: LDA, NMF, BERTopic		Augmented Reality Filters	Overlays showing identity or solidarity	Seen in videos/selfies during crises
	Stance Detection	Detects ideological or political position	Useful for policy-related framing or moderation		AI-Generated Content	Synthetic images or deepfakes	Requires detection in misinformation mitigation
Behavioral Signals	Posting Patterns	Frequency, time gaps, intensity	May reflect distress, trauma, or migration stages	Real-World Application	Missing Migrant Detection	Combine hashtags, comments, and image metadata	Supports crowd-traced sightings
	Deactivated/Suspended Accounts	Sudden disappearance from platforms	Early warning of risk or suppression		Digital Ethnography	Language and narrative evolution	Reveals coping, grief, or solidarity
	Story/Status Updates	Time-limited posts	Often overlooked, critical in real-time analysis		Border Event Monitoring	Live stream monitoring on TikTok, Instagram	Useful in detecting pushbacks or gatherings
Psychosocial Dynamics	Emoji Usage	Emotional tone or cultural expression	Trackable across crisis phases (e.g., 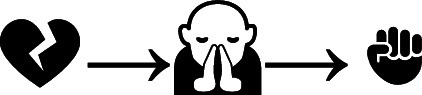 )		Misinformation Detection	Identify reused images/videos across events	Reverse search and repost tree analysis
	Language Switching	Multilingual code-switching	Relevant in diasporic and multilingual settings				
	Narrative Shift Over Time	Change in tone or focus	Suitable for autoethnography or longitudinal analysis				

Digital signals such as pinned posts, live streams, or spatiotemporal patterns can indicate urgency, assist in route reconstruction, and provide semantic insights. Media content provides visual evidence for verification, while often-overlooked signals like emoji usage or “last seen” notifications can reveal psychological states or presence patterns. Practical applications encompass digital ethnography, border incident surveillance, and crowdsourced notifications, illustrating the role of varied digital signals in humanitarian efforts and scholarly investigations of migrant disappearances.

### Humanitarian data infrastructure framework

**Figure 1.**
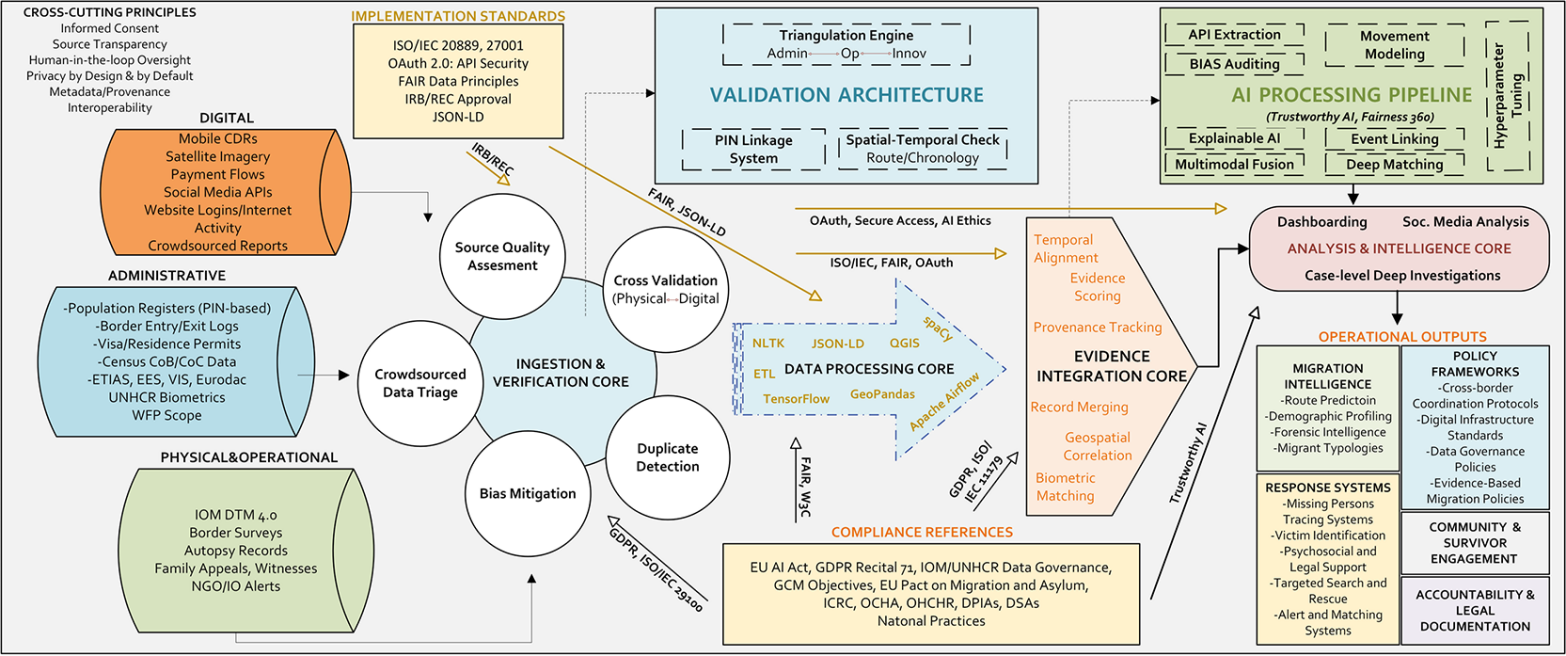
Architecture of Missing Migrants System.

The proposed framework integrates diverse digital data sources into a systematic ingestion-processing-analysis workflow as given in Figure 1. Ingestion encompasses the acquisition of data from structured databases, unstructured social media streams, and sensor networks. Data processing involves the cleaning, normalization, and enrichment of data to guarantee interoperability among various formats and platforms. Analysis employs both computational and qualitative methods to produce actionable insights, with ethics-integrated governance integrated at every stage to ensure alignment with humanitarian principles. Consent and privacy protocols reduce harm and build trust. This modular framework adjusts to various operational contexts, resource levels, and technological capacities, an essential characteristic in migration scenarios characterized by rapid changes.

As outlined in Table 3, digital traces are categorized into ten domains (e.g., content, location, semantics, media). Examples range from multilingual posts and live-stream comments indicating urgency to geospatial markers for route reconstruction and network graphs for tracing and rumor monitoring. Semantic methods, including sentiment analysis and stance detection, elucidate ideological and emotional dynamics. Concurrently, visual content combined with metadata facilitates identity verification and the identification of disinformation. The classification ( Table 2) and structured methodology ( Table 3) collectively establish a replicable pathway for ethically grounded, evidence-based research on missing migrants.

**Table 3.** The Humanitarian data structure framework.

Core	Purpose (based on scientific literature)	Implementation standards
Ingestion & Verification	To collect, verify, and validate data from diverse sources (social media, NGOs, governments, families) while ensuring legal compliance and minimizing harm.	ISO/IEC 27001, GDPR, IRB/REC Approval, OAuth 2.0 (if APIs used)
Data Processing Core	To clean, anonymize, transform, standardize, and enrich data (text, image, geospatial) for further integration and analysis.	ISO/IEC 20889, FAIR Data Principles, JSON-LD
Evidence Integration Core	To link, reconcile, and structure multimodal data (e.g., witness accounts, satellite images, autopsy reports) into coherent case records or event chains.	ISO/IEC 11179, JSON-LD, FAIR, W3C Linked Data & PROV Standards
Analysis & Intelligence Core	To derive actionable insights, detect patterns, support identification, and produce early warning intelligence or policy input.	GDPR (AI Profiling Limits), EU Ethics Guidelines for Trustworthy AI, OAuth 2.0 (API security)
Cross-layer Controls	To ensure that all processes (across all cores) adhere to shared principles like privacy, transparency, ethics, interoperability, and security. These controls govern the entire pipeline and guarantee trust, legal compliance, and user protection.	Informed Consent, GDPR (Art. 5&25), ISO/IEC 27001&27701- W3C PROV, IRB/REC Approval, Privacy by Design, FAIR, Human-in-the-loop Governance

### Core components: Data ingestion and verification core

The Data Ingestion and Verification Core functions as the system's primary entry point, tasked with the secure acquisition, authentication, and initial organization of data from various sources, including governmental systems (e.g., border logs, biometric registries), humanitarian organizations, digital platforms, and impacted communities. It employs real-time validation via blockchain provenance tracking and algorithmic trust ratings, ensuring GDPR compliance with differential privacy ( Kenny et al., 2021). Ethical precautions encompass dynamic consent mechanisms and automated ethical review workflows that adhere to WHO’s digital health governance principles, mitigating the dangers associated with participatory data misuse.

Key roles encompass metadata tagging utilizing descriptors like language, geolocation accuracy, and thematic categories to provide semantic interoperability and traceability, in alignment with FAIR principles. Duplicate detection employs probabilistic matching, flagging uncertain cases for human review to maintain integrity and dignity (Asylum Capacity Support Group, 2024). Human-in-the-loop verification is vital for validating open-source and community content ( CIDOC, 2025).

By incorporating ethical supervision and data governance from the outset, this core guarantees that only verifiable, contextually relevant information flows into the system, following principles like do-no-harm, informed consent, and survivor-centered design ( OCHA, 2025; OHCHR, 2025). Ingestion systems, metadata extraction libraries, and validation frameworks with contextual categorization engines are among tools that can help with this stage.

### Data processing core

Following ingestion, the Data Processing Core transforms raw inputs into organized, machine-actionable datasets using multimodal preprocessing processes. It processes textual, visual, geographic, and metadata inputs often acquired in time-sensitive and emotionally charged situations to ensure that disparate sources are standardized, enriched, and ready for downstream analysis. Core activities include cleansing, pseudonymization, and normalization of dates, coordinates, and naming schemas. NLP methods are utilized on unstructured text.

Algorithms for image and signal processing identify objects, improve low-resolution images, and, where allowed, conduct facial analysis. Geospatial tagging and reverse geocoding facilitate spatial analysis, including clustering and route reconstruction. Metadata production facilitates traceability and auditability, especially in legal or forensic scenarios. Reports are classified into categories such as SOS alert or confirmed missing to support triage and response. Multimodal alignment integrates varied content, connecting social media posts with satellite imagery and field reports, to provide precise case timelines and individual data linkages. Semantic harmonization is accomplished using JSON-LD/RDF mappings to CIDOC-CRM ontologies and Schema.org extensions, guaranteeing interoperability while maintaining contextual integrity.

Despite these capabilities, ethical problems remain, such as algorithmic bias, contextual loss from anonymization, and the potential of overprocessing user-generated content. Nonetheless, the results such cleaned datasets, geocoded event logs, and enriched multimodal records, feed directly into the Evidence Integration and Analysis Cores, allowing for predictive modeling, dashboard construction, and dignified humanitarian action.

### Evidence integration core

The core constitutes the foundational framework of the missing migrants information system, converting disparate, multimodal data into cohesive case records. This function is crucial in humanitarian and forensic situations, because information regarding persons is fragmented across biometric registries, satellite imagery, social media posts, witness testimonies, and autopsy reports. As a forensic module, it merges fragmented data into verifiable, ethical narratives.

At its core is entity resolution, which connects records that pertain to the same individual or event via probabilistic and rule-based matching of names, biometrics, spatiotemporal metadata, and narrative indicators. Multimodal matching integrates text, photos, geolocation, and physiological data for a holistic perspective ( Chambers et al., 2021). Spatiotemporal linkage reconstructs timelines and migratory trajectories, frequently employing graph neural networks (GNNs) to describe disappearance dynamics ( Zhang et al., 2023). Record merging diminishes fragmentation, whereas evidence scoring allocates confidence levels based on reliability, match strength, and temporal consistency, facilitating priority in humanitarian operations.

Provenance tracking guarantees transparency, reproducibility, and legal compliance using W3C PROV-O-compliant chains and blockchain-based notarization, resulting in OHCHR-compliant disappearance event chains aligned with FAIR and data justice principles ( UNICRI, 2025). De-duplication resolves redundant reports, such as aliases for the same individual, by metadata harmonization in accordance with ISO/IEC 11179 standards.

Erroneous matches risk misidentification and family distress, while managing biometric and geolocation data raises privacy concerns, especially post-mortem. The core implements supervision, auditability, and transparency measures by utilizing tools such as Neo4j or RDF-based knowledge graphs for data linking, OpenRefine or Dedupe.io for record alignment, and Bayesian networks with uncertainty quantification for probabilistic linkage. These methods enhance precision, accountability, and transparency in intricate humanitarian investigations.

### AI processing pipeline

Recent advances extend traditional text and image analysis to ubiquitous digital traces such as “last seen” indicators, read receipts, and typing notifications, which provide subtle yet powerful signals of presence or disappearance. Shifts in emoji usage can reflect community sentiment, while voice-based media offer narratively rich, emotionally expressive data for computational ethnography and emotion recognition ( Santhanam et al., 2019; Düvell, 2024; Zarei et al., 2024). In migrant and diasporic communities, memes, GIFs, and augmented reality filters communicate identity, solidarity, and political stances ( Zhang et al., 2025).

The AI pipeline ( Table 4) transforms data into actionable intelligence through a structured process:

• Feature Engineering: Extracting key attributes (e.g., geolocation, emotional cues, visual patterns).
• Modeling: Employing task-specific architectures like CNNs for image recognition and transformers (e.g., BERT) for NLP.
• Validation & Tuning: Training models against verified ground truth data and optimizing them via hyperparameter tuning.
• Deployment: Integrating models into operational systems (APIs, dashboards) for automated high-risk alerting.
• Maintenance: Ensuring ongoing reliability through monitoring, drift detection, and iterative feedback.

**Table 4.** AI Processing Pipeline.

Module/Trend	Description	Application/Novelty
CLIP-based Image-Text Retrieval	Match visual social media content (e.g., missing person image) to textual descriptions	Enables visual confirmation and deep matching across modalities
Video Analysis with Whisper + Vision	Use OpenAI’s Whisper for speech-to-text alongside vision transformers (e.g., Flamingo) to analyze videos	Extracts movement clues and distress signals from audiovisual content
Geo-aware LLMs (GeoLLM, SpatialBERT)	Enhance geolocation accuracy in language models by grounding place references	Disambiguates similarly named locations (e.g., “Paris, Texas” vs. “Paris, France”)
Trajectory Extraction from Text	Apply NLP to reconstruct migration journeys from social narratives (e.g., “left Tripoli, seen near Lampedusa”)	Enables spatiotemporal modeling of migrant pathways
Displacement Pattern Detection	Identify recurring mobility behaviors in user posts over time and space	Supports early detection of crisis dynamics such as border shifts or camp evacuations
Narrative Arc Detection	Detect temporal story progression within user-generated content	Tracks transformation from “missing” to “found” or “mourned,” useful for humanitarian analysis
Cultural Code-Switch Detection	Analyze shifts in language, tone, or emoji use across multilingual/diasporic users	Illuminates identity negotiation and cultural adaptation in digital refugee discourse
Digital Kinship Modeling	Use textual and network analysis to infer social or familial relations	Facilitates family tracing and contextual case understanding
Bias Mitigation in Migration Narratives	Detect representational imbalances or stereotypical framing in media or posts	Strengthens ethical oversight and fairness in data-driven outputs
Participatory Annotation Tools	Enable NGOs or affected communities to review or label data and outputs	Supports human-in-the-loop governance and ethical co-production of knowledge
Explanation Generator (e.g., LIME, SHAP for LLMs)	Provide interpretable justifications for model decisions (e.g., why a post was flagged)	Enhances transparency and trust, especially in high-stakes or policy-related uses
Close + Distant Reading Hybrid	Combine LLM-powered distant reading with close textual analysis for digital humanities	Supports macro (corpus-level) and micro (case-level) interpretations of migrant discourse
Affective Computing	Analyze how emotions such as grief, solidarity, or trauma are expressed online	Goes beyond polarity sentiment analysis, focusing on emotional nuance
AI-Generated Synthetic Narratives for Simulation	Use LLMs to simulate migrant testimonies for scenario testing or misinformation stress-testing	Helps evaluate AI sensitivity and narrative bias under different conditions
Historical Archive Integration	Link real-time AI pipelines with historical data (e.g., ICRC records, oral histories)	Enriches longitudinal analysis and connects past and present migration trajectories

Explainability is vital in sensitive scenarios ( EU, 2025). Tools such as SHAP and LIME make model outputs interpretable, while ethical principles from the EU’s Trustworthy AI framework and IOM’s Data Responsibility Principles reinforce justice, accountability, and human dignity. Technical implementation relies on Apache Kafka and Airflow for orchestration, TensorFlow Data Validation for preprocessing, MLflow and Kubernetes for deployment, FastAPI for monitoring, and visualization platforms for real-time decision-making.

AI poses disinformation risks, including deepfakes and synthetic media. Current applications include triangulating geolocation data from metadata, hashtags, and comments to locate missing persons ( Gillespie et al., 2018); analyzing meme diffusion and language shifts to study solidarity and coping mechanisms ( Pink et al., 2015); monitoring platforms for signs of crowd movements or state violence ahead of official reports; and detecting misinformation through reverse image searches and reposting pattern analysis ( Zubiaga et al., 2019). As AI increasingly engages with multimodal, ephemeral, and emotionally charged data, methodological rigor and ethical sensitivity become indispensable. The pipeline balances technical scalability with accurate representation of displaced populations’ lived experiences, vulnerabilities, and rights.

### Analysis and intelligence core

The core operates as the analytical engine of the missing migrants data infrastructure, converting validated inputs into actionable intelligence for humanitarian response, policy formulation, risk mitigation, and accountability. It draws on crisis informatics and data science, integrating computational, statistical, and human-in-the-loop methods to identify patterns, anticipate hazards, and simulate complex scenarios. Its essential functions include identifying spatiotemporal trends like high-risk routes and employing predictive models with transformer-based architectures to forecast the probability of future disappearances or dangerous crossings.

Analytical functions include GIS-based clustering, route reconstruction, and heatmapping to locate disappearances in time and space. Social network analysis investigates the relationships between migrants, smugglers, and humanitarian players using metadata and digital footprints, while temporal chronologies rebuild event sequences to formulate traceable case narratives. Text and image analysis applies NLP to testimonies and uses object or facial recognition on photos and satellite imagery, augmented by deep learning technologies. Insights are shared through real-time OCHA-compliant dashboards. Risk scoring and alert systems identify anomalies, such as abrupt increases in disappearance reports, and provide prompt warnings consistent with humanitarian early warning protocols.

Ethical AI safeguards are embedded across the system. This encompasses adversarial debiasing, community-driven validation, and adherence to the EU’s Ethics Guidelines for Trustworthy AI and the IOM’s Data Ethics Principles. Governance controls strengthen these measures via zero-trust frameworks, algorithmic consequence analysis, rights-impact evaluations and survivor-led audit committees ( Fischer et al., 2022). By using anti-oppressive design principles, the system guarantees transparency, equity, and respect for dignity. The outputs such as predictive models and visual analytics facilitate evidence-based policymaking, enhance humanitarian response, and promote preventive efforts for migrant disappearances.

### Operational outputs: policy-relevant migration intelligence

The system’s analytical capabilities generate operational outputs across five interconnected domains that support a comprehensive humanitarian response to migrant disappearances: migration intelligence, response systems, policy frameworks, community engagement, and accountability through legal documentation.

Migration intelligence synthesizes multi-source data into a strategic decision-support tool. Its core functions include:

• Route Prediction: Applying statistical and ML models to forecast migration flow shifts.
• Demographic Profiling: Identifying high-risk groups (e.g., unaccompanied minors, stateless persons) to guide interventions.
• Forensic Intelligence: Linking DNA, autopsy, and post-mortem data with missing persons databases under strict ethical safeguards.
• Spatio-Temporal Analysis: Mapping high-risk areas, seasonal patterns, and event-driven disappearances.
• Digital Signal Analysis: Using phone metadata, social media behavior, and remittance flows as proxies for real-time mobility and distress.

This integrated understanding of patterns, vulnerabilities, and outcomes enables proactive resource allocation and policy formulation.

Response system translates intelligence into coordinated field interventions grounded in humanitarian law and human rights principles. This system can guide search and rescue operations, facilitate identification, and provide assistance to families. It also connects disappearance reports with active search efforts, integrating biometric data, witness testimonies, and field observations through interoperable systems. Similarly, the ICRC's Trace the Face initiative exemplifies this model by enabling families to upload photos and supporting cross-border searches ( ICRC, 2025).

As an output of the system, victim identification combines forensic and digital methods including DNA analysis, autopsies, personal belongings, and AI-based facial recognition under strict legal protocols such as Interpol’s Disaster Victim Identification standards and UN recommendations. Family notification mechanisms deliver timely, sensitive updates with robust privacy protections to prevent misuse, crucial for families facing stigma or legal risk. Real-time tools, including early warning systems, automated alerts, and coordination platforms, integrate predictive analytics with geospatial intelligence to identify high-risk migration corridors and enable rapid deployment of resources ( UN PULSE, 2025). Community engagement through participatory validation, survivor-led audits, and culturally sensitive communication fosters trust, improves data quality, and ensures appropriate interventions. Together, these systems transform data into humanitarian action, linking analysis with field dignity.

Evidence-based policy frameworks guide national and international responses to migrant disappearances, ensuring interventions are legally sound, ethical, and aligned with protection standards. These frameworks address border governance, identification protocols, and familial rights to information and redress. By preserving verifiable data trails and forensic linkages, they uphold accountability, support victim identification and repatriation, and enable the prosecution of abuses. Ultimately, these outputs advance a rights-based approach that integrates technological innovation with legal rigor, community participation, and ethical accountability.

### Cross-layer controls

Cross-layer controls mitigate risks during ingestion, processing, and dissemination, ensuring that humanitarian data systems react promptly while preserving dignity, privacy, and minimizing harm. These safeguards -rooted in FAIR principles, informed consent, anonymization, and context-sensitive access protocols- function horizontally throughout the architecture to enhance trust, accountability, and resilience. Their efficacy relies on robust multi-stakeholder governance, ongoing risk evaluation, and proactive community involvement. Digital infrastructures for identifying and locating missing migrants depend not only on essential tasks such as intake, processing, analysis, and integration, but also on comprehensive systems that ensure ethics-integrated governance, data integrity, and legal compliance throughout the data lifecycle.

Informed consent is a fundamental precaution. Families, witnesses, and migrants must comprehend the utilization, storage, and dissemination of their information. In situations involving trauma or loss, consent must be explicit, culturally appropriate, sustained over time, and revocable, adhering to international human rights and humanitarian data ethics norms. Ethical oversight enhances this process, generally via Institutional Review Boards or Research Ethics Committees, which evaluate hazards associated with data collection, algorithmic processing, and disclosure. In migratory contexts, such vigilance is crucial to avert stigmatization, the exposing of undocumented individuals, or the retraumatization of families.

Auditability requires comprehensive logging, lineage tracking, and documentation of assumptions and metrics to ensure trust and compliance with FAIR and OECD guidelines. Interoperability, achieved through standards like JSON-LD, RDF, and ISO/IEC 11179, enables semantic and technical alignment across organizations. Privacy is maintained via encryption, access controls, data minimization, and advanced techniques like differential privacy, adhering to ISO/IEC 27001 and 20889. User accountability is enforced through tiered permissions and audit trails. Collectively, these embedded controls form the ethical and operational foundation, ensuring technological innovation is matched by institutional responsibility and resilience.

## 5. Discussion

This architecture integrates multimodal digital traces with analytical pipelines and embedded ethical safeguards, aligning with studies on digital platforms in crisis response ( W3, 2025). Consistent with studies that used crowdsourced data for earthquake damage assessment or flood detection, the current architecture shows how unconventional data sources such as social media posts, geolocation metadata, and ephemeral online signals, can provide actionable intelligence in contexts of heightened humanitarian risk ( Li et al., 2023; Han et al., 2024)

This work integrates diverse inputs through entity resolution and multimodal fusion. It utilizes probabilistic and machine learning approaches to reconstruct timelines, migration trajectories, and disappearance dynamics, similar to previous initiatives that have integrated diverse records for crisis mapping and forensic investigation ( Xu et al., 2023; Gintova, 2019). The architecture applies transformer models and graph neural networks to reveal risk patterns, consistent with prior research on geospatial and temporal reconstruction ( Mızrak, 2024; Ellouze et al., 2025). The integration of explainable AI techniques and stringent validation approaches addresses current discussions on transparency, accountability, and trust in algorithmic systems utilized in humanitarian operations ( Uluğ et al., 2023; Theja Bhavaraju et al., 2019).

Ethical considerations are fundamental to system design. Prior studies on digital humanitarianism have highlighted the risks linked to misinformation, surveillance, and privacy violations in the utilization of crisis data ( Han et al., 2024; Kovras and Robins, 2016). This study incorporates adversarial debiasing, community-driven validation, and rights-based audit mechanisms, thereby aligning with international principles of data responsibility and ethical AI ( Walk et al., 2023; Zhou et al., 2025). Provenance tracking and de-duplication functions effectively tackle operational challenges in humanitarian data integration, as redundant or unverifiable information often compromises efficiency and credibility ( Gintova, 2019).

Despite these advancements, specific limitations require attention. Previous research indicate that data representativeness poses a challenge due to the uneven distribution of social media activity across people and geographies, resulting in potential analytical blind spots ( Talabi et al., 2022). Entity resolution techniques pose the possibility of false positives, while biases inherent in training datasets may perpetuate systemic imbalances. Furthermore, the incorporation of sensitive biometric and geolocation information exacerbates ethical and privacy issues, especially in critical humanitarian and post-mortem inquiries. The system is modular and scalable but requires validation in field deployment. Future research must emphasize pilot implementations in partnership with NGOs, intergovernmental organizations, and impacted communities to assess practicality, ethical precautions, and policy implications in practical contexts.

## 6. Implementation and expected value

As the proposed framework is designed as a scalable and modular sociotechnical infrastructure, it is expected to be implemented through collaborative initiatives between humanitarian informatics researchers, forensic experts, and digital ethnographers. The research community can utilize the novel typology of 32 digital trace types to standardize the collection of decentralized evidence, moving beyond isolated data types like text to include ambient signals such as "last seen" indicators and psychosocial markers like emoji shifts. The proposed modular architecture, consisting of ingestion, processing, evidence integration, AI, and analysis cores, is designed to be adopted by scholars and humanitarian actors as a scalable and methodologically reproducible framework for the rigorous validation of digital traces in migration research. By leveraging interoperable standards like JSON-LD and CIDOC-CRM mappings, researchers can ensure that their technical artifacts are compatible with international human rights and FAIR data principles.

The framework addresses critical structural deficiencies in current migratory data infrastructures, specifically the "informational blind spots" found in fragmented migration routes. By integrating multimodal traces that span from geospatial markers to emotionally expressive multilingual appeals, the system enables a more robust and fine-grained spatiotemporal reconstruction of migrant trajectories. Unlike traditional border registers that often fail to capture the full scope of disappearances, this method offers a "bridge" between computational innovation and rights-based governance. Furthermore, the inclusion of stringent safeguards such as dynamic consent and explainable AI promotes "Data Justice," ensuring that technical advancements do not come at the expense of participant dignity or privacy.

## 7. Conclusion

The proposed framework enhances humanitarian data science by demonstrating the systematic transformation of fragmented and multimodal digital traces into cohesive and ethically regulated evidence streams. The approach guarantees that technical innovation is paired with institutional accountability by incorporating safeguards like explainable AI, provenance tracking, and survivor-led supervision. It contributes to continuing discussions regarding openness, accountability, and resilience in humanitarian informatics.

This study emphasizes the capacity of computational, statistical, and human-in-the-loop methodologies to generate actionable insights that facilitate humanitarian responses, guide policy, and advance preventive measures for migrant disappearances across every stage of the migration cycle. Future efforts must prioritize collaboration and survivor-led validation to guarantee that data infrastructures are both technically scalable and socially legitimate, inclusive, and responsive to the actual experiences of displaced and vulnerable populations.

Two significant limitations exist. First, evolving platform affordances, content norms, and access regimes require longitudinal benchmarking and cross-platform replication. Second, the scarcity of verifiable ground truth data complicates validation. Addressing this necessitates institutionalized partnerships with NGOs and forensic authorities to obtain verified labels and establish survivor-centered feedback. Future work should prioritize piloting domain-specific multilingual models, co-designing evaluation metrics that balance precision/recall with harm-aware measures (e.g., false-alert burden on families), and conducting prospective trials to assess impacts on triage time, identification rates, and notification quality.

## Ethics and consent

Ethical approval and consent were not required.

## Data Availability

No data is associated with this article.
