# Humanitarian data infrastructures for missing migrants: A multimodal and ethics-integrated framework

**DOI:** 10.12688/openreseurope.23124.2

**Published:** 2026-06-17

**Authors:** M. Fevzi Esen, Ecenur Aydemir

**Affiliations:** 1 Department of Health Information Systems, Hamidiye Institute of Medical Sciences, University of Health Sciences, Istanbul, Turkey; 2 Department of Healthcare Management, Faculty of Health Sciences, Acibadem Mehmet Ali Aydinlar University, Istanbul, Turkey

**Keywords:** Missing migrants; migration governance; sociotechnical systems; digital traces; humanitarian informatics; early warning systems.

## Abstract

Migrant disappearances have become a critical humanitarian crisis on global scale. However, the lack of strong, consolidated data across irregular migration routes is a barrier to effective intervention. In this paper, we aim to employ a sociotechnical systems perspective to design an ethics-infused infrastructure. Our primary objective is to convert multimodal digital traces into actionable intelligence, thereby supporting search, identification, and policy accountability. To standardize the analysis of decentralized digital evidence, we define a typology of 32 trace types encompassing ten domains, categorized from geospatial markers to psychosocial signals. This classification is the basis for the modular architecture of five linked cores. We present a novel approach that combines entity resolution and spatiotemporal reconstruction with provenance tracking and explainable AI to support effective humanitarian action. These features enable robust early warning capabilities and provide decision support aligned with OCHA standards. The framework aims to facilitate compliance with FAIR data principles and international human rights standards through embedding cross-layer safeguards including dynamic consent, auditability and interoperability. This research provides a scalable, replicable method to validate digital evidence throughout the migration cycle. It bridges computational innovation and rights-based governance. Future work should focus on operational pilots to assess feasibility, triage efficiency, identification accuracy, and family notification protocols.

## 1. Introduction

The disappearance of migrants is a critical global concern. According to Missing Migrants Project, the International Organization for Migration (IOM) has documented more than 75,000 missing persons along migration routes since 2014 ( IOM, 2025). Traditional systems like border registers and forensic databases often fail to capture the full scope of these incidents due to fragmented surveillance, reporting delays, and geopolitical constraints ( Obinna, 2025). With the expansion of digital connectedness, migrants and their communities are progressively producing real-time, socially embedded traces such as geotags, multilingual appeals, and visual evidence on platforms like X, Facebook, and TikTok ( Wilkinson and Castaneyra-Ruiz, 2021). Despite their potential, these traces are under-reported because of unstructured formats, ethical concerns, and institutional resistance ( Wahid et al., 2025). As a result, families experience continuous underreporting issues, knowledge gaps, and prolonged uncertainty.

Digital platforms produce a variety of traces, including posts, hashtags, geotags, and interaction. They also provide time-sensitive, socially embedded signals that may complement official sources. The difficulty of transforming heterogeneous, multilingual, and emotional based data into operational intelligence remains to lack typologies, interoperable workflows, and ethics-integrated governance. While social media analytics has been investigated in the context of disaster response and refugees, a holistic framework for irregular migrant disappearances is still missing. Current research focuses on individual data types and does not include the combination of multimodal traces and ethical governance ( Leasure et al., 2023). This gap prevents the development of field-ready systems based on dignity, privacy and accountability. To address these challenges, this study follows a Sociotechnical Systems (STS) perspective. It aims to conceptualize humanitarian data infrastructures as environments where technical items, institutional policies and human actors intersect. This framework considers digital traces as socially situated signals, not neutral inputs, and investigates the whole “migration cycle” from the initial distress in transit to the legal identification of missing persons.

To ensure conceptual clarity throughout this methodology, we define key terms as follows: Digital traces are deliberately or inadvertently produced digital footprints (e.g., posts, geotags), while ambient traces are passive, background signals of digital presence (e.g., “last seen” statuses, typing indicators). Humanitarian data infrastructures refer to the sociotechnical ensemble of hardware, software, legal protocols, and human actors that process these traces. The goal of this infrastructure is to produce actionable intelligence, which is validated, OCHA-aligned outputs formatted for immediate field use. Finally, the system is governed by data justice (fairness in how vulnerable people are made visible and treated by data systems) and survivor-led governance (decision-making processes that formally center affected families in auditing and oversight).

The primary methodological contribution of this paper is an integrated sociotechnical framework for a modular humanitarian data infrastructure to address the fragmented surveillance and ethical governance gaps identified above. This article proposes an unified methodological approach to the handling of decentralised digital evidence throughout the migration cycle, rather than the separation of the issues of data categorisation, technical processing and ethical governance.

To implement this general conceptual framework, the paper is structured around three foundational sub-components:

Foundational typology: Proposing a new typology of 32 different types of digital traces across 10 domains to standardise the collection of multimodal signals.

1. Technical architecture and AI pipeline: Proposing a five-core modular pipeline (ingestion, preprocessing, evidence integration, AI and analysis) to show how these traces can be systematically integrated.
2. An ethics-integrated governance model: Incorporating data stewardship, privacy and dynamic consent principles into the technical architecture to ensure compliance with Data Justice and human rights standards. This study presents these elements as an integrated methodological framework, providing a scalable blueprint for researchers and organisations to ethically validate and integrate digital traces into humanitarian response systems.

In particular, this study is organized around three research questions:

1. How to systematically categorize the diverse digital and ambient traces to assist in the investigation of missing migrants?
2. What is the architectural framework and AI pipeline to ethically integrate multimodal traces with institutional records?
3. How can data justice, survivor-led governance and rights-based safeguards be operationalized in this technical pipeline?

In this study, Section 2 presents the current humanitarian and migratory data infrastructures and investigates the possibilities of digital traces as additional evidence. Section 3 discusses the theory basis in sociotechnical systems and data justice. Section 4 provides a detailed description of the proposed typology and modular architecture. Finally, the paper highlights the governance challenges and policy implications required for the safe integration of these technologies into global humanitarian practice.

## 2. Background

Humanitarian responses to missing migrants relied on institutional data systems, including border control records, missing persons registries, forensic archives, and international databases. However, the literature indicates that structural barriers remain:

• Spatiotemporal fragmentation: There are significant gaps in isolated, high-risk areas, such as coastal routes and desert crossings, resulting in underreporting and “informational blind spots” ( UNSD, 2025).
• Procedural delays: Bureaucratic processes, inconsistent reporting, and geopolitical constraints restrict the exchange of information ( Calderón et al., 2024; Tefera and Gamlen, 2025).
• Identification failures: Limited physical evidence, delays in notifications and restricted access to official systems leave many missing persons unidentifiable. Projects are not resilient to these challenges, as they are based on fragmented and retrospective statistics provided by NGOs and official bodies ( Black, 2024). Such restrictions show that more data is needed in real time.

### Digital traces as complementary humanitarian sensors

Research in digital migration studies indicates that social media enables decentralized solutions to crises ( Rejali and Heiniger, 2020; Leurs & Ponzanesi, 2024; Sithole, 2024). Unlike conventional infrastructures, digital platforms enable near-real-time data acquisition, decentralized validation, and rapid support network mobilization ( Erokhin and Komendantova, 2024; Herrera et al., 2025). Crowdsourcing during emergencies and large-scale digital humanitarianism show the ability of community-oriented platforms to generate actionable intelligence ( Dave, 2017; Poblet et al., 2018).

Migrants use social media to seek help, plan routes, and stay connected ( Talabi et al., 2022). This activity generates both intentional posts and contextual “ambient” traces such as geotags, network structures, and cross-platform interaction patterns that indirectly indicate mobility, community building, and vulnerability ( Walk et al., 2023). Social media, as a reactive “social sensor”, helps detect migration distress signals ( Theja Bhavaraju et al., 2019).

Mobile phone metadata and internet searches can be used to estimate migration flows, intentions, and integration ( Tjaden, 2021). For example, mobile phones are essential for navigation on migration routes, but they also expose users to surveillance and misinformation risks ( Dekker et al., 2018).

### Analytical frameworks for heterogeneous data

Analyzing multiple types of digital traces requires frameworks that can combine different modalities into actionable insights. AI techniques are essential for classifying crisis-related content, analyzing sentiments, and recognizing user roles ( Rabani et al., 2023). Topic modeling tools are also useful for finding new topics in large text corpora, and spatio-temporal analysis techniques are useful in route mapping, needs clustering and defining effect zones ( Kankanamge et al., 2020; Villodre and Criado, 2020). Recent developments (e.g., Joint Spatio-Temporal Topic-Sentiment framework and Topic2Labels) provide scalable and multimodal methodologies that reduce the dependence on manually annotated datasets. However, the effectiveness depends on the adaptation of models to the linguistic and cultural contexts of migration ( Hanny and Resch, 2025; Deng et al., 2020).

### Governance, ethics, and responsible implementation

Despite advances in analytical capability, structural constraints continue to prevent the ethical and operational integration of digital trace analytics into missing migrant studies. These challenges fall into four inter-related areas:

• Ethical issues (e.g., algorithmic bias, surveillance exposure);
• Technical constraints (e.g., limitations of NLP, interoperability issues);
• Governance limitations (e.g., no consent protocols, institutional opposition);
• Methodological limitations (e.g., over-dependence on text-based data, ignoring ambient traces).

These issues increase the vulnerabilities of migrants and weaken trust and data utility. Table 1 summarizes these complex challenges and presents the proposed framework as an integrated approach.

**Table 1.** Multidimensional challenges and humanitarian implication.

Challenge domain	Key issues	References
Ethical risks	Surveillance exposure, algorithmic bias (e.g., Western-centric NLP models), voyeurism, re-traumatization	Leasure et al., 2023 ; Spijkerboer, 2023; Jørgensen, 2024; OCHA, 2025; OHCHR, 2025; UNICRI, 2025
Technical limitations	Low-resource language support, interoperability gaps, misinformation proliferation	Gillespie et al., 2018 ; Santhanam et al., 2019; Rabani et al., 2023; Düvell, 2024
Institutional barriers	Restrictive data policies, bureaucratic resistance to crowdsourcing, legal ambiguities	De Hoon et al., 2019 ; Bélanger and Candiz, 2019; Mohamed et al., 2020; Haller and Yanaşmayan, 2023
Methodological gaps	Over-reliance on single data types (e.g., Twitter text), neglect of ambient/emergent traces (e.g., AR filters, voice notes)	Kreutzer et al., 2025 ; Zhang et al., 2025; Zhou et al., 2025

The FAIR principles and comprehensive humanitarian ethics frameworks are still inadequately employed ( Wilkinson et al., 2016). Limited research integrates dignity-centered governance measures such as permission processes, bias audits, and provenance tracking across the data lifecycle ( Mohamed et al., 2020; Kreutzer et al., 2025). This study aims to develop a comprehensive typology of digital data sources ranging from geotags to ambient traces and map them within a humanitarian data infrastructure framework. The proposed framework fills these gaps with embedded cross-layer controls like algorithmic auditing and dynamic consent mechanisms. This also combines methodological innovation and ethics-integrated governance to improve timeliness, localization and inclusivity in humanitarian action, while safeguarding dignity, privacy and accountability.

## 3. Theoretical framework: Data justice and sociotechnical humanitarianism

The ethical and technical design of humanitarian data infrastructures requires more attention than functional innovation. It requires a critical perspective on the role of such systems in shaping power relations, inclusion and legitimacy in humanitarian settings. The article constructs its framework through three related domains of research: sociotechnical systems theory, critical data studies, and data justice in humanitarian technology. Theoretical perspectives shape and regulate this infrastructure, influencing how data is ingested, modeled, and governed. These principles are embedded in the pipeline’s design through modularity, transparency, and survivor-led mechanisms for validation and oversight.

### Sociotechnical systems in humanitarian contexts

Humanitarian technologies are embedded within institutions, cultures, and power relations that shape the production, interpretation, and use of data. This interplay is underlined by the notion of sociotechnical systems, suggesting that technologies are created and are operated within the boundaries and rules of their environments ( Kitchin, 2022). For crisis response, this means data is never separated from the social and political forces surrounding it. This duality is reflected in digital traces that are exchanged through platforms such as WhatsApp or Instagram. They may provide a potential insight into displacement events, but are also influenced by the dynamics of the platform, the linguistic choices, and the structure of the network. They lose their social meaning when treated as neutral inputs. The design of a humanitarian data infrastructure needs to respond to these embedded social dynamics.

Digital traces shared through platforms like WhatsApp or Instagram illustrate this dual nature. While they provide potential insight into displacement events, they are also shaped by platform dynamics, linguistic choices, and network structures. Treating them as neutral inputs strips them of social meaning. In designing a humanitarian data infrastructure, responsiveness to these embedded social dynamics becomes necessary. Taylor (2021) highlights several models that can enable distributed oversight and ethical flexibility. These priorities are reflected in the modular pipeline architecture discussed in this study.

### Critical data studies and the politics of traces

Traces are not static records. They are created and selected and sometimes ignored to comply with the priorities and assumptions of the systems processing them. Critical data studies offers methods to investigate how this happens, particularly for populations who fall outside formal systems of registration or legal recognition ( Iliadis & Russo, 2016).

Data collection infrastructures for migrants often operate under conditions of uncertainty and fragmented access. In this space, signals like “last seen” timestamps or fragmented message histories can become crucial for finding someone, but their significance is always contextual. Grohmann (2025) conceptualize data infrastructures as contested spaces where recognition, authority, and interpretation are negotiated. According to Hoffmann (2020), systems often try to include marginalized subjects while relying on unexamined frameworks for classifying information. Because these underlying structures are not questioned, these well-intentioned systems can end up reproducing harm. This framework includes mechanisms for trace provenance, pathways for consent, and feedback loops for these dynamics. The system understands traces as being located in ethics and context. They are not just evidence to be checked or rejected. They are inputs in a wider investigative process that are socially meaningful.

### Data justice and the ethics of humanitarian AI

Data justice provides a method to assess the actual functioning of humanitarian systems. This concept shifts the emphasis away from technical performance or privacy to broader concerns around agency, representation and accountability ( Taylor, 2017; Heeks & Renken, 2018). This is particularly relevant where algorithmic systems are used to process sensitive or ambiguous information, such as signals relating to disappeared individuals.

Recent research emphasized the importance of reflexivity and transparency in algorithmic governance. Srivastava (2023) notes the need for systems that can manage doubt and external evaluation in decision-making under uncertainty, especially in high-stakes scenarios. Within this framework, modularity contributes to this goal by enabling adaptation across institutional contexts while maintaining ethical consistency. The system’s architecture enables different implementations by forensic experts, humanitarian organizations or family-led groups, while retaining auditability and explainability.

Madianou (2019) argues that the lens of justice, rather than efficiency or scalability, should be used to evaluate humanitarian innovation. In response, this framework includes participatory governance tools that enable annotation, feedback and redress by those affected and their communities. Ethical oversight is not a secondary concern but a fundamental component running throughout the data lifecycle.

### Ethical traceability and system design

The typology of different trace types within the framework is not only a technical mapping, but also an epistemological structure. Traces are historically and ethically located. These are not isolated signals but components of broader systems of risk, and recognition. Such dynamics align with Milan and Treré’s (2020) analysis of how marginalized populations navigate datafication through resistance, improvisation, and situated knowledge production. The system is not based on maximum visibility or full data integration, but on trace integrity, modular governance and flexible implementation. The system is built on rights-based guidance, including OHCHR’s (2021) framework for data use in humanitarian settings. Every module in the pipeline is designed to support contextual reasoning, accountability and trace-level review.

## 4. Results

### Derivation and the Validation of the Typology

This section categorizes digital data sources that are relevant to the investigation of missing migrants, in particular from social and digital media.

The literature for identifying potential trace types is mainly based on three fields: crisis informatics, digital ethnography, and humanitarian data science. Data categories are derived through a hybrid process, utilizing deductive methods to identify established constructs within existing disaster communication and digital migration studies ( Reuter & Kaufhold, 2018; Leurs & Smets, 2018). An inductive method is used to observe emerging, ambient traces (such as emoji shifts, augmented reality filters, and meme propagation) used by migrant networks during transit and crisis ( Highfield & Leaver, 2016).

Inclusion criteria required a trace type to be: (1) empirically observable on digital platforms used by migrants or their families; (2) able to provide spatiotemporal, forensic, or psychosocial context; and (3) technically extractable (even if computationally difficult). For overlapping categories (e.g., a geotagged photo contained visual evidence and technical metadata), traces were assigned to the category that best served their primary functional humanitarian utility (e.g., the image was assigned to “Media Content” for visual verification, and the coordinates to “Interaction/Location”). To ensure operational relevance, the draft was reviewed by domain experts in multidisciplinary discussions within international working groups on migrant disaster victim identification (MDVI) and with input from health information management, data science, and forensic investigation. The classification was then validated against existing humanitarian workflows to ensure that it was aligned with the operational realities of the ICRC Trace the Face initiative and Interpol’s Disaster Victim Identification (DVI) protocols.

Sources are classified into thematic categories covering specific data types in Table 2. These categories reflect the complexities of digital traces in migration.

**Table 2.** Data sources in missing migrants research.

Trace category	Specific data types	Humanitarian & analytical utility	Methodological notes
Content (Text)	Posts, captions, comments, auto-translated texts, pinned posts	Core for NLP, sentiment tracking, and studying cross-lingual flows.	Multilingual models required; pinned posts signal high-priority urgency.
Media (Visual)	Photos, embedded videos, AI visual recognition tags, Alt Text	Visual verification, terrain recognition, and facial matching.	Reverse image search essential for misinformation detection.
Interaction & Location	Conversation threads, live streams, geotags, inferred NLP locations	Tracks discourse, spatiotemporal mobility, and last-known locations.	Geoparsing tools extract text-based places; requires longitudinal linking.
Technical Metadata	Timestamps, platform proxies, edit histories	Reconstructs precise event timelines and detects manipulated narratives.	Often stripped by platforms; requires API or data donation access.
Networks & Virality	Mention networks, repost trees, crowdsourced alerts (#WhereIsX)	Enables kinship tracing, activist mapping, and rumor tracking.	Crucial for coordinating community-based search efforts.
Ambient Traces	Last seen online, typing statuses, read receipts	High-value presence detection (e.g., WhatsApp).	Requires explicit family data donation; cannot be publicly scraped.
Psychosocial Dynamics	Emoji usage, narrative shifts, voice/audio notes	Encodes cultural expressions, emotional states, and trauma.	Emotionally rich but highly subjective; requires digital ethnography.
Experimental Traces	Memes, GIFs, AR filters, AI-generated synthetic media	Illuminates identity, solidarity, and political resistance.	High risk of deepfakes; requires advanced synthetic media detection.

Digital signals (e.g., pinned posts, live streams, and spatiotemporal patterns) can indicate urgency, help reconstruct routes, and provide semantic insights. Furthermore, the accompanying media content provides visual proof for verification. Signals like emoji trends or last seen notifications can disclose mental states or presence trends. Digital ethnography, monitoring border incidents and crowdsourced alerts are some of the real world applications, illustrating how varied digital signals inform humanitarian initiatives and academic research on migrant disappearances.

### Trace-risk matrix

Digital traces vary fundamentally in their accuracy and ethical vulnerability. Consequently, a failure to differentiate between these data types introduces substantial risks for the integrity.

To operationalize humanitarian data principles ( OHCHR, 2021) and address the valid concerns regarding digital migration records ( Tjaden, 2021; Dekker et al., 2018), Table 3 introduces a Trace-Risk Matrix, which systematically categorizes data based on its accuracy and potential for harm. The matrix assesses important categories from the typology along seven dimensions: evidentiary value, ethical sensitivity, consent requirements, reliability, platform accessibility, risk of harm, and suitability for automated AI processing.

**Table 3.** Trace-risk matrix for missing migrant digital evidence.

Trace category	Evidentiary value	Ethical sensitivity	Consent requirement	Reliability	Platform accessibility	Risk of harm	Automation suitability
Public Content	Low to Medium (leads/context)	Low to Medium	Implicit (Publicly broadcast)	Variable (High risk of misinformation)	High (Open APIs, Scrapeable)	Low (if de-identified)	High (NLP, Topic modeling)
Media/ Visual	High (Verification)	High	Explicit (if used beyond original context)	Medium to High (requires metadata verification)	Medium (Subject to platform takedowns)	High (Facial exposure, surveillance risk)	High (Computer Vision, Object Detection)
Ambient/Technical	Medium (Presence/Timeline)	High	Explicit (Requires user/family provision)	High (System-generated)	Low (Locked behind E2E encryption or private UI)	High (Breaches private communication expectations)	Low to Medium (Requires specific data donation)
Psychosocial	Low (Contextual/Emotional)	Medium to High	Context-dependent	Low (Highly subjective, culturally encoded)	Medium	Medium (Risk of misinterpretation or bias)	Medium (Requires culturally tuned NLP/Audio models)
Institutional/ Forensic	Absolute (Identification)	Very High	Explicit/Legal Mandate	Very High	Very Low (Restricted government/NGO databases)	Extreme (Deportation, legal targeting)	High (Secure entity resolution systems)

### Data accessibility and ethical collection tiers

A fundamental sociotechnical reality of humanitarian data infrastructures is that technical extractability does not equate to ethical or legal legitimacy. The framework recognizes that digital traces are affected by various platform architectures, terms of service and privacy expectations. Therefore, to meet the technical feasibility and legal compliance, the framework classifies all data sources into six different levels of accessibility:

1. Public/Open Data: This includes openly broadcast social media content (e.g., public hashtags, crowdsourced alerts, and open-source spatial intelligence). Although these data are technically accessible via automated scraping, they require processing and anonymization. This is essential to prevent surveillance exposure and safeguard vulnerable populations, consistent with international human rights data guidelines ( OHCHR, 2021).
2. Platform API-Accessible Data: Aggregated search trends, semantic shifts, or generalised network topologies are available through official developer endpoints. This data is subject to platform-specific rate limits, terms of service, and algorithmic shifts and is thus collected under corporate data sharing principles and not open access.
3. Voluntarily Submitted Family/Community Data (Data Donation): Psychosocial and environmental markers (e.g., “last seen online” status, typing indicators, private voice notes and direct messages) may not be collected routinely. Within this framework, they are strictly categorized as voluntary submissions requiring explicit informed consent. This is increasingly operationalised through “data donation” methodologies, proposed by Boeschoten et al. (2022), in which families voluntarily contribute localised exports of communication histories to support specific investigative efforts without breaking platform encryption.
4. NGO or Institutional Case Records: This tier consists of operational data including border interaction logs, demographic profiles from organisations (e.g., UNHCR, family appeals). Their integration depends on formalised data-sharing agreements, strict access controls and purpose-limitation protocols, similar to governance models of the ICRC’s Trace the Face initiative ( ICRC, 2025).
5. Sensitive Restricted Data: The tier contains highly regulated forensic data for DVI (e.g., biometrics, DNA profiles and autopsy records). This data processing is limited to authorised forensic entities only, and requires secure, encrypted interoperability standards (e.g., ISO/IEC 27001).
6. Data Not Recommended for Routine Collection: Some digital signals carry risks that exceed their humanitarian benefits. Routine or automatic collection of cross-platform interaction histories, scraping of algorithmic boosting metrics associated with specific user identities, or tracking of account deactivations without user consent is not recommended. These practices not only breach platform’s terms of service and sidestep user consent but also generate the same surveillance risks that already put marginalized groups at a disadvantage.

### Humanitarian data infrastructure framework

**Figure 1.**
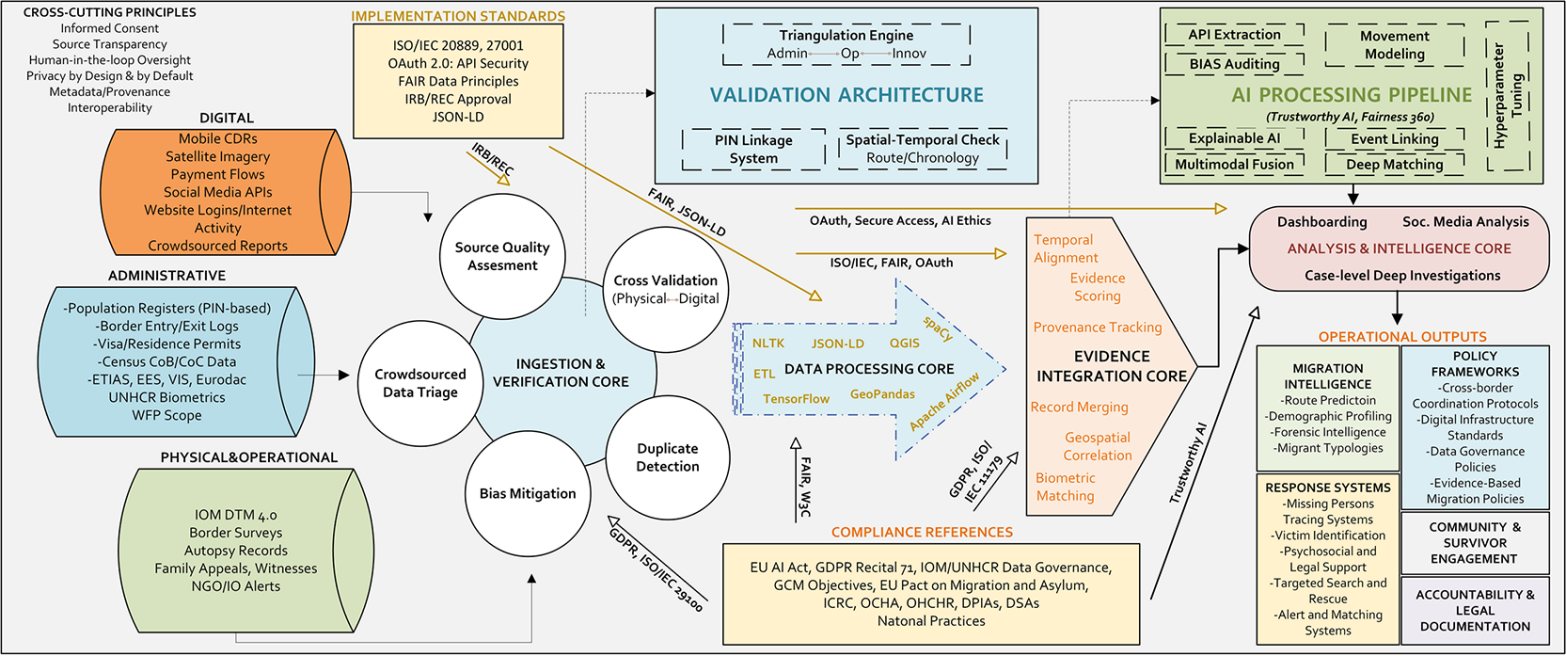
Architecture of Missing Migrants System.

Figure 1 shows a framework that systematically ingests, processes and analyzes various digital data sources. Ingestion includes extracting data from structured databases, unstructured social media streams, and sensor networks. Data processing involves cleaning, normalization and enrichment of data to ensure interoperability between different formats and platforms. Analysis employs computational and qualitative approaches to generate actionable insights while ethics-integrated governance is embedded at each stage to achieve alignment with humanitarian principles. Consent and privacy protocols reduce harm and build trust. This modular framework adapts to various operational contexts, resource levels, and technical capacities. Such flexibility is essential when navigating the rapid, unpredictable nature of migration scenarios.

Digital traces are categorized into ten domains ( Table 2). Examples include multilingual posts and live-stream comments that suggest urgency, geospatial markers for reconstructing routes, and network graphs for tracing and rumor monitoring. Semantic approaches like sentiment analysis and stance detection are useful for understanding ideological and emotional dynamics. At the same time, visual content along with metadata allows identity verification and disinformation identification. The classification ( Table 2) and structured methodology ( Table 4) collectively constitute a replicable framework for ethically grounded, evidence-based research on missing migrants.

**Table 4.** The Humanitarian data structure framework.

Core	Purpose (based on scientific literature)	Implementation standards
Ingestion & Verification	To collect, verify, and validate data from diverse sources while ensuring legal compliance and minimizing harm.	ISO/IEC 27001, GDPR, Institutional Review Board (IRB) or Research Ethics Committee (REC) Approval, OAuth 2.0 (if APIs used)
Data Processing Core	To clean, anonymize, transform, standardize, and enrich data for further integration and analysis.	ISO/IEC 20889, FAIR Data Principles, JSON-LD
Evidence Integration Core	To link, reconcile, and structure multimodal data into coherent case records or event chains.	ISO/IEC 11179, JSON-LD, FAIR, W3C Linked Data & PROV Standards
Analysis & Intelligence Core	To derive actionable insights, detect patterns, support identification, and produce early warning intelligence or policy input.	GDPR (AI Profiling Limits), EU Ethics Guidelines for Trustworthy AI, OAuth 2.0 (API security)
Cross-layer Controls	To ensure that all processes adhere to shared principles like privacy, transparency, ethics, interoperability, and security. These controls govern the entire pipeline and guarantee trust, legal compliance, and user protection.	Informed Consent, GDPR (Art. 5&25), ISO/IEC 27001&27701- W3C PROV, IRB/REC Approval, Privacy by Design, FAIR, Human-in-the-loop Governance

### Core components: Data ingestion and verification core

Data Ingestion and Verification Core functions as the system’s primary entry point. This manages secure acquisition, authentication, and organization of disparate data streams ranging from border logs and biometric registries to humanitarian and community-sourced digital traces. The system employs real-time validation through blockchain-based provenance tracking and algorithmic trust ratings. These mechanisms ensure strict GDPR compliance by integrating differential privacy into the data architecture ( Kenny et al., 2021). Ethical safeguards encompass dynamic consent processes and automated ethical review procedures aligned with WHO’s digital health governance principles, addressing risks linked to participatory data misuse.

The framework uses metadata descriptors (e.g., language, geolocation precision, and thematic categories) to tag content. This process enables the semantic interoperability and traceability required by the FAIR principles. Duplicate detection uses probabilistic matching and uncertain cases are flagged for human review to preserve integrity and dignity ( Asylum Capacity Support Group, 2024). Human-in-the-loop verification is critical to validate open source and community content ( CIDOC, 2025). This core is designed to ensure that only verifiable and contextually relevant information is sent to the system through the application of ethical supervision and data governance at the outset, according to principles such as do-no-harm, informed consent and survivor-centered design ( OCHA, 2025; OHCHR, 2025).

### Data processing core

Following ingestion, Data Processing Core transforms raw inputs into organized, machine-actionable datasets using multimodal preprocessing processes. The system processes textual, visual, geographic, and metadata inputs, which are often collected in time-sensitive and emotionally charged situations. This ensures that multiple data sources are standardized, enriched, and prepared for downstream analysis. Core activities include cleansing, pseudonymization, and normalization of dates, coordinates, and naming schemas.

Algorithmic image and signal processing techniques are employed to detect objects and enhance image resolution. Where explicitly authorized, this core also perform facial analysis. Spatial analysis is enabled by geospatial tagging and reverse geocoding. In legal and forensic scenarios, systematic production of metadata is essential for ensuring robust traceability and auditability. Reports are classified into categories such as SOS alert or confirmed missing to support triage and response. Multimodal alignment combines different content, linking social media posts to satellite images and field reports, to provide accurate timelines of cases and connections of individuals. The system utilizes JSON-LD/RDF mappings to align data with CIDOC-CRM ontologies and Schema.org extensions. This process of semantic harmonization ensures both system interoperability and the preservation of contextual integrity.

However, these capabilities come with ethical issues (e.g., algorithmic bias, contextual loss from anonymization), and the risk of overprocessing user-generated content. These processes yield cleaned datasets, geocoded event logs, and enriched multimodal records. These outputs feed directly into the Evidence Integration and Analysis Cores to support predictive modeling, dashboard construction, and dignified humanitarian action.

### Evidence integration core

This core is the backbone of the missing migrants information system. It converts heterogeneous multimodal data into unified case records. This task is important for humanitarian and forensic applications where individual information is scattered across biometric databases, satellite imagery, social media, witness statements and autopsy reports. As a forensic module, it integrates sparse data into verifiable and ethical narratives.

The framework relies on entity resolution, which uses a combination of probabilistic and rule-based matching to link records referring to the same person or event. By incorporating text, photos, geolocation, and physiological data, the system achieves a holistic view that goes beyond single-source analysis ( Chambers et al., 2021). Spatiotemporal linkage reconstructs timelines and migratory trajectories, often using graph neural networks (GNNs) to model disappearance dynamics ( Zhang et al., 2023). Record merging reduces fragmentation, and evidence scoring assigns confidence levels according to reliability, match strength and temporal consistency, thus allowing for prioritization in humanitarian operations.

Provenance tracking provides a framework for transparency and reproducibility by using W3C PROV-O-compliant chains and blockchain notarization. This allows for the construction of disappearance event chains that meet OHCHR requirements while remaining consistent with FAIR and data justice principles ( UNICRI, 2025). Deduplication addresses duplicate reports (e.g., aliases for the same person) by harmonizing metadata according to ISO/IEC 11179 standards.

Mismatches may lead to misidentification and family distress. Privacy issues are raised concerning the handling of biometric and geo-location data especially beyond the post-mortem stage. The core architecture implements supervision, auditability and transparency measures. The framework enhances precision and accountability in sophisticated humanitarian investigations by exploiting knowledge graphs (e.g. Neo4j or RDF) for data linking and tools such as OpenRefine and Dedupe.io for record alignment, and Bayesian networks with uncertainty quantification for probabilistic matching.

### AI processing pipeline

Recent research expands the scope of analysis beyond textual and visual data to include digital signals such as “last seen” indicators, read receipts, and typing notifications. These signals may provide powerful signals regarding an individual’s presence or disappearance. Shifts in emoji usage can reflect community sentiment, while voice-based media offer narratively rich, emotionally expressive data for computational ethnography and emotion recognition ( Santhanam et al., 2019; Düvell, 2024; Zarei et al., 2024). Memes, GIFs, and augmented reality filters communicate identity, solidarity, and political positions within migrant and diasporic communities ( Zhang et al., 2025).

AI pipeline transforms data into actionable intelligence through a structured process ( Table 5):

• Feature Engineering: Extracting key attributes,
• Modeling: Employing task-specific architectures,
• Validation and Tuning: Training models against verified ground truth data and optimization via hyperparameter tuning,
• Deployment: Integrating models into operational systems for automated high-risk alerting.
• Maintenance: Ensuring ongoing reliability through monitoring, drift detection, and iterative feedback.

**Table 5.** AI processing pipeline.

Module/Trend	Description	Application/Novelty
CLIP-based Image-Text Retrieval	Match visual social media content (e.g., missing person image) to textual descriptions	Enables visual confirmation and deep matching across modalities
Video Analysis with Whisper + Vision	Use OpenAI’s Whisper for speech-to-text alongside vision transformers (e.g., Flamingo) to analyze videos	Extracts movement clues and distress signals from audiovisual content
Geo-aware LLMs (GeoLLM, SpatialBERT)	Enhance geolocation accuracy in language models by grounding place references	Disambiguates similarly named locations (e.g., “Paris, Texas” vs. “Paris, France”)
Trajectory Extraction from Text	Apply NLP to reconstruct migration journeys from social narratives (e.g., “left Tripoli, seen near Lampedusa”)	Enables spatiotemporal modeling of migrant pathways
Displacement Pattern Detection	Identify recurring mobility behaviors in user posts over time and space	Supports early detection of crisis dynamics such as border shifts or camp evacuations
Narrative Arc Detection	Detect temporal story progression within user-generated content	Tracks transformation from “missing” to “found” or “mourned,” useful for humanitarian analysis
Cultural Code-Switch Detection	Analyze shifts in language, tone, or emoji use across multilingual/diasporic users	Illuminates identity negotiation and cultural adaptation in digital refugee discourse
Digital Kinship Modeling	Use textual and network analysis to infer social or familial relations	Facilitates family tracing and contextual case understanding
Bias Mitigation in Migration Narratives	Detect representational imbalances or stereotypical framing in media or posts	Strengthens ethical oversight and fairness in data-driven outputs
Participatory Annotation Tools	Enable NGOs or affected communities to review or label data and outputs	Supports human-in-the-loop governance and ethical co-production of knowledge
Explanation Generator (e.g., LIME, SHAP for LLMs)	Provide interpretable justifications for model decisions (e.g., why a post was flagged)	Enhances transparency and trust, especially in high-stakes or policy-related uses
Close + Distant Reading Hybrid	Combine LLM-powered distant reading with close textual analysis for digital humanities	Supports macro (corpus-level) and micro (case-level) interpretations of migrant discourse
Affective Computing	Analyze how emotions such as grief, solidarity, or trauma are expressed online	Goes beyond polarity sentiment analysis, focusing on emotional nuance
AI-Generated Synthetic Narratives for Simulation	Use LLMs to simulate migrant testimonies for scenario testing or misinformation stress-testing	Helps evaluate AI sensitivity and narrative bias under different conditions
Historical Archive Integration	Link real-time AI pipelines with historical data (e.g., ICRC records, oral histories)	Enriches longitudinal analysis and connects past and present migration trajectories

In sensitive humanitarian contexts, explainability is not merely technical but essential ( EU, 2025). To ensure this, tools such as SHAP and LIME are employed to make model outputs interpretable, while ethical frameworks (e.g., the EU’s Trustworthy AI guidelines, IOM’s Data Responsibility Principles) serve to uphold justice, accountability, and human dignity. This synthesis of ethics and transparency is supported by a comprehensive technical infrastructure, ranging from Apache Kafka and Airflow for orchestration to MLflow and Kubernetes for deployment. Finally, monitoring is maintained via FastAPI, with visualization platforms providing the interface for real-time decision-making.

The dual nature of AI presents both challenges, and opportunities for humanitarian inquiry. These are being explored across several key domains:

• Search and Rescue: Triangulating geolocation via metadata and social markers to locate missing persons ( Gillespie et al., 2018),
• Social Resilience: Analyzing meme diffusion and linguistic shifts to map coping mechanisms and solidarity ( Pink et al., 2015),
• Crisis Monitoring: Tracking crowd movements and state violence prior to official reports,
• Disinformation Detection: Utilizing reverse image search and pattern analysis to verify claims and combat misinformation ( Zubiaga et al., 2019).

### Analysis and intelligence core

The core operates as the analytical engine of the missing migrants data infrastructure. It converts validated inputs into actionable intelligence for humanitarian response, policy formulation, risk mitigation, and accountability. It is based on crisis informatics and data science, integrating computational, statistical and human-in-the-loop approaches to identify patterns, forecast hazards and model complex scenarios. The key ones are: (i) detecting the spatiotemporal trends such as high-risk routes; and (ii) using predictive models (with transformer-based architecture) to predict the probability of future disappearances or dangerous crossings.

Analytical functions include GIS-based clustering, route reconstruction, and heatmapping to locate disappearances in time and space. Social network analysis utilizes metadata and digital footprints to examine the complex relationships between migrants, smugglers, and humanitarian actors. Simultaneously, temporal chronologies are used to reconstruct sequences of events, generating traceable case narratives. Additionally, text and image analysis leverages natural language processing (NLP) to parse documentation, while deep learning models perform object and facial recognition on photographic and satellite imagery. Real-time insights are shared through OCHA-compliant dashboards. Risk scores and alert systems detect anomalies (e.g., a sudden spike in reports of disappearances), and provide timely warnings in line with humanitarian early warning protocols.

Ethical AI safeguards are integrated into the system. This includes adversarial debiasing, community-driven validation and compliance with the EU’s Ethics Guidelines for Trustworthy AI and the IOM’s Data Ethics Principles. Governance controls enhance these measures through zero-trust models, algorithmic consequence analysis, rights-impact assessments, and survivor-led audit committees ( Fischer et al., 2022). The implementation of anti-oppressive design principles is central to promoting transparency, equity, and respect for human dignity. This ethical framework empowers the system’s outputs to drive evidence-based policymaking.

### Operational outputs: policy-relevant migration intelligence

For operational clarity, the framework distinguishes the output of the framework according to the end user: For humanitarian purposes, the focus is on real-time search, rescue and family alerts. For forensic purposes, the use is limited to post-mortem disaster victim identification (DVI). For research purposes, anonymized data is used for longitudinal digital ethnography. For policy purposes, macro level intelligence is produced for OCHA-aligned accountability frameworks.

The system’s analytical capabilities generate operational outputs across five interconnected domains that support a comprehensive humanitarian response to migrant disappearances: migration intelligence, response systems, policy frameworks, community engagement, and accountability through legal documentation.

Migration intelligence is a strategic decision support tool, which integrates multi-source data. Such integrated understanding of patterns, vulnerabilities and outcomes may enable the allocation of resources and the formulation of policies proactively. Its principal functions are:

• Route Prediction: Performing statistical and ML models to forecast migration flow shifts.
• Demographic Profiling: Identifying high-risk groups (e.g., unaccompanied minors, stateless persons) to guide interventions.
• Forensic Intelligence: Linking DNA, autopsy, and post-mortem data with missing persons databases under strict ethical safeguards.
• Spatio-Temporal Analysis: Mapping high-risk areas, seasonal patterns, and event-driven disappearances.
• Digital Signal Analysis: Using phone metadata, social media behavior, and remittance flows as proxies for real-time mobility and distress.

Response system transforms intelligence in coordinated field interventions based on humanitarian law and human rights principles. The framework operationalizes search and rescue efforts by linking disappearance reports with live search data via an interoperable pipeline of biometric information, witness reports, and field observations. This integration directly assists in identification and family reunification. The model aligns with established initiatives such as the ICRC’s “Trace the Face,” which leverages cross-border, family-initiated image analysis to facilitate the tracing of missing persons ( ICRC, 2025).

The system synthesizes forensic evidence (e.g., DNA, autopsy records, and personal effects) with AI-based facial recognition to facilitate victim identification. All procedures are executed in accordance with legal mandates, including Interpol’s DVI standards and UN guidelines.

The framework balances sensitive communication with large-scale data coordination. On one hand, notification mechanisms ensure that families receive critical updates with the privacy safeguards necessary to mitigate risks of stigma or legal vulnerability. On the other, the integration of predictive analytics and geospatial intelligence empowers real-time coordination, allowing for the identification of high-risk corridors and the accelerated deployment of humanitarian support ( UN PULSE, 2025). Community engagement through participatory validation, survivor-led audits, and culturally sensitive communication fosters trust, improves data quality, and ensures appropriate interventions. Together, these systems transform data into humanitarian action, linking analysis with field dignity.

Evidence-based policy frameworks guide both national and international responses to migrant disappearances, ensuring that all interventions are legally sound, ethical, and aligned with international protection standards. These standards deal with border governance, protocols for identification, and familial rights to information and redress. A key component of accountability, victim identification and prosecution of human rights violations is the maintenance of verifiable data trails and forensic linkages. It supports a rights-based approach that combines technical innovation with legal scrutiny, participatory design and robust ethical oversight.

### Operationalizing ethical governance and rights-based safeguards

Cross-layer controls mitigate risks throughout the data lifecycle by ensuring a rapid, responsive approach within humanitarian systems. These measures are essential for maintaining the dignity and privacy of individuals while systematically minimizing the potential for harm. Beyond core functional tasks, digital infrastructures for migrant identification require robust governance architectures. Ensuring data integrity and legal compliance necessitates the integration of ethical imperatives at every stage of the data lifecycle. According to Kreutzer et al. (2025), the ethical challenges surrounding autonomy, non-maleficence, beneficence, and justice are significantly deteriorated when algorithmic systems interact with vulnerable groups during humanitarian crises. Thus, through concrete and enforceable mechanisms adapted to the complexities of migrant disappearances, this framework implements its cross-layer controls based on FAIR principles.

***Consent, proxy agency, and withdrawal***

Informed consent is a vital safeguard that requires participants to understand the usage, storage, and dissemination of their data. In traumatic contexts, this necessitates a consent process that is clear, culturally appropriate, dynamic, and revocable. However, the process of obtaining informed consent is very complicated when the data subject is missing, deceased or detained. The system implements this through a delegated proxy model. If a migrant cannot be reached, consent can only be given by verified next-of-kin or legally recognized humanitarian representatives, in the vital interest of the subject (GDPR Art. 6(1)(d) and 9(2)(c)).

Furthermore, consent is treated as a revocable state. Families are provided with secure, low-barrier channels to withdraw their data. Upon withdrawal, a “deletion cascade” is triggered across the architecture, removing the data from active processing pipelines, except where overriding legal mandates (e.g., active forensic disaster victim identification investigations) temporarily suspend withdrawal requests. IRBs or RECs can provide ethical oversight of these processes to prevent stigmatization, disclosure of undocumented persons, or retraumatization of families.

***Firewalls, access tiering, and misuse prevention***

In accordance with ISO/IEC 27001 and 20889, privacy is maintained through encryption, granular access controls, and differential privacy. The system architecture integrates strict purpose limitation to further protect family submitted data from external surveillance. A key operational requirement is to prevent mission creep: humanitarian data is firewalled from law enforcement and border agencies. Therefore, the combination of zero-trust data architectures and formal Memorandum of Understanding (MoUs) acts as a legal and technical barrier to the repurposing of humanitarian data for immigration enforcement or deportation.

Tiered Role-Based Access Control (RBAC) ensures accountability by restricting data access to the specific requirements of each stakeholder, ranging from macro-level insights for authorities to sovereign data control for families and exclusive biometric access for forensic experts. This framework is underpinned by standardized interoperability protocols (e.g., JSON-LD, RDF, and ISO/IEC 11179), ensuring seamless and secure alignment between partner organizations. Given the cross-border context of international migration, all data exchanges are governed by Standard Contractual Clauses (SCCs) and IOM’s Data Protection Manual.

***Lifecycle, auditing, and redress mechanisms***

Accountability is embedded in the data lifecycle. On the retention side, the framework does not allow for indefinite storage but sets a temporal scope. Ambient traces and crowdsourced alerts are immediately deleted if they are found to be irrelevant after the initial triage. Active cases are kept on record until identity verification or family reunification is established. The system must be carefully tracked on the lineage and all algorithmic results must be documented in detail according to the FAIR and OECD standards. Technical transparency is achieved through tamper-evident audit logs that comply with W3C PROV and link every action in the system to a verified credential. This technical accountability is underpinned by a robust human-rights framework: families can access formal remedies, including appeals to Data Protection Officers (DPOs). Independent, survivor-led ethics boards could be empowered to initiate algorithmic audits or even suspend operations. The system may ensure that technical innovation is grounded in institutional responsibility and ethical accountability.

### Reproducible workflow

Humanitarian data systems are technological archives, not reproducible workflows. To ensure technical soundness, replicability and compliance with the ethical constraints described in previous sections, the proposed architecture is not a monolithic application, but a stratified workflow. The system implementation should be divided into four distinct operational tiers based on technical need and ethical risk.

• Required Minimum Components: The integrity and security of the entire framework depend on foundational ingestion, deduplication, and triage processes, which together provide the essential operational architecture for secure data management.
• Optional Analytical Extensions: Advanced computational modules (e.g., NLP-driven thematic clustering or semantic analysis) that enhance intelligence but are not strictly required for base-level case management.
• High-Risk Components Requiring Special Approval: Modules that process Tier 5 biometric data or automate entity resolution. Due to the severe consequences of misidentification, these components cannot operate without explicit legal mandates and dual-authorization mechanisms.
• Future Research Components: Experimental systems (e.g., predictive mobility modeling and synthetic narrative stress-testing) that are not sufficiently validated with ground-truth for active field deployment.

Table 6 provides an operational matrix to facilitate field deployment and replication by international working groups and humanitarian organizations. For each module, it specifies the inputs, outputs, governing standards, decision rules, validation checks, and human actors responsible who need to be in the loop.

**Table 6.** Operational workflow and component categorization.

Component tier & module	Inputs	Outputs	Standards	Exemplary decision rule	Validation check	Responsible actors
1. Required Minimum	Tier 1 (Open Data) & Tier 4 (NGO records); User-submitted case files.	Cleaned, normalized datasets; Immutable audit logs; Flagged duplicates.	ISO/IEC 27001; W3C PROV; FAIR.	If source provenance is unverified or ambiguous, quarantine data and flag for human review.	Human-in-the-loop manual review of quarantined data; Automated duplicate hashing.	Humanitarian Data Stewards; IT Administrators.
2. Optional Extension	Multilingual text, interaction metadata (Tiers 1 & 2), donated ambient traces (Tier 3).	Thematic clusters; Spatiotemporal trajectory estimations; Sentiment scoring.	JSON-LD; Schema.org extensions.	If NLP language confidence score < 85%, halt automated translation and route to manual queue.	Cross-translation verification via native-speaking digital ethnographers.	Data Scientists; Digital Ethnographers.
3. High Risk	Tier 5 Forensic data (DNA, autopsies); Geolocation coordinates; High-res facial images.	Probabilistic case match scores; Victim identification narratives.	GDPR (Art. 9); Interpol DVI protocols; ICRC guidelines.	Any algorithmic match over 90% confidence requires mandatory dual-authorization before generating a notification alert.	Multi-stakeholder ethics board review; Post-mortem forensic expert sign-off.	Forensic Experts; Designated DPOs; Medical Authorities.
4. Future Research	Historical displacement trajectories; Synthetic AI-generated test data.	Early-warning vulnerability forecasts; Route disruption models.	EU AI Act (Sandbox compliance).	System outputs must be strictly watermarked and siloed as “Simulated/Advisory” and cannot influence active active search/rescue.	Ground-truth benchmarking against retrospective data; Algorithmic bias audits.	Health Informatics Researchers; Academic Working Groups.

Table 7 presents a structured Trace Mapping, Standards, and Validation Matrix to provide a granular operational blueprint for field deployment. This matrix maps each primary trace category to its practical application. For every category, it details the governing legal or technical standard, the main ethical vulnerability, and the required validation protocol. This mapping ensures that any technical replication aligns with the framework’s cross-layer controls.

**Table 7.** Trace mapping, standards, and validation matrix.

Trace category	Example trace	Primary source	Legal/Technical standard	Primary ethical risk	Validation requirement
Public Content	Crowdsourced alerts (#)	Open APIs (e.g., X, TikTok)	FAIR, GDPR Art. 6(1)(e)	Voyeurism, Surveillance	Human-in-the-loop (HITL) triage verification.
Media/Visual	Geotagged images	Open Web/User Uploads	ISO/IEC 27001	Misinformation/Deepfakes	Reverse image search and metadata auditing.
Ambient/Technical	Read receipts/Timestamps	Encrypted Messaging Apps	GDPR Art. 9, E2E Encryption	Privacy breach	Explicit proxy consent via “Data Donation” protocols.
Institutional Data	NGO Border Logs	UNHCR/ICRC	ICRC Data Protection	Mission creep (Law enforcement access)	Formal Data Sharing Agreements (MoUs).
Forensic Data	Biometrics/Autopsy	Gov. Databases/Medical	Interpol DVI/ISO 20889	Post-mortem dignity violation	Mandatory Ethics Board/Legal mandate review.
Psychosocial	Emoji shifts/Language	Public/Private text	EU Trustworthy AI Guidelines	Algorithmic cultural bias	Survivor-led qualitative evaluation (Digital Ethnography).
Network Data	Interaction/Mentions	Social Graphs	W3C PROV (Provenance)	Unintended exposure of undocumented networks	Cryptographic anonymization prior to processing.

### Pilot scenario: A case-based application

To illustrate the practical operation of the proposed framework without the ethical concerns of using real sensitive data, this section introduces a synthetic pilot scenario. This hypothetical example illustrates how the interconnected cores of the architecture and dedicated AI pipeline modules process disparate data into actionable intelligence, while applying established ethical safeguards.

***Phase 1: Ingestion & verification core (Proxy Consent & Triage)***

The workflow begin when a humanitarian NGO receives a family report of a missing person (“Subject M”). The family provides a recent picture of themselves and a stated intended route. The picture is used by the NGO that employs the proxy agency protocols of the Cross-layer Controls of the system to trigger a public social media appeal (Tier 1 data). Meanwhile the Data Ingestion and Verification Core starts to seek open-source channels to match digital traces, recording all intake via W3C PROV standards.

***Phase 2: Data processing core & ai processing pipeline (Feature Extraction)***

Within 48 hours, the ingestion system detects a scraped, geotagged post on a public platform with an image and text. AI Processing Pipeline is active. The system analyzes the visual image in the post using the CLIP-based Image-Text Retrieval module, confirming 88% biometric similarity to Subject M. Meanwhile, NLP models in the Data Processing Core parse a witness comment under the post: “I saw him yesterday heading toward the inland border crossing.” The modules of the Geo-aware LLMs (SpatialBERT) and Trajectory Extraction from Text attempt to convert this narrative into a physical route.

***Phase 3: Evidence integration core (Anomaly Detection)***

The system attempts to match the scraped post with the institutional case record for Subject M. During the spatiotemporal linkage process, the Evidence Integration Core identifies significant conflicting location information: the geotag in the metadata places the post near a coastal transit hub, but the AI-extracted trajectory from the witness comment indicates an inland route.

***Phase 4: Cross-layer controls (Human Review & Explainable AI)***

To avoid false positive identifications, the architecture’s decision rules explicitly prohibit automated entity resolution in the presence of spatial anomalies. In instances where data consistency cannot be verified, the system suspends the merge process and flags the conflict for manual review by a human analyst, ensuring that ambiguous records do not bypass verification. A digital ethnographer accesses the data. The reviewer employs the Explanation Generator (e.g., LIME/SHAP) of the AI pipeline to analyze how the model weighted the text. The reviewer notes that the witness comment aligns with known bot-driven misinformation patterns. The reviewer manually overrides the text-based location and validates the metadata geotag. Each decision is then immutably logged to ensure a transparent audit trail.

***Phase 5: Analysis & intelligence core (Scoring and Output)***

With the conflict resolved, the system calculates a probabilistic case confidence scoring. Factoring in the facial match, the validated geotag, and temporal proximity, the system assigns a score of 82%. Because this falls below the stringent 90% High-Risk threshold required for automated definitive identification, the intelligence core does not issue a final match notification. Instead, it triggers the generation of a humanitarian action report. This OCHA-compliant intelligence brief is distributed to field rescue teams, designating the coastal transit hub as a high-probability search area, successfully translating digital traces into an evidence-based field intervention, while maintaining investigative integrity.

### Validation and harm-aware evaluation metrics

The proposed architecture includes XAI tools (e.g., SHAP and LIME) but model interpretability does not automatically ensure model validity, fairness or safety. Emerging literature on XAI in high-stakes domains highlights the risk that algorithmic explanations can inadvertently foster automation bias or overwhelm the user, ultimately failing to provide actionable accountability ( Rudin, 2019; Kaur et al., 2020). In humanitarian settings, algorithmic “performance” cannot be simply defined as computational speed or isolated accuracy, but should prioritize the core principle of “do no harm” and safeguard human dignity and equity ( Coppi et al., 2019). The framework is used for highly sensitive processes such as early warning and family notification and thus requires a pre-defined validation plan prior to deployment in the field. The system should be evaluated in future pilot implementations on three interrelated domains: algorithmic validity, operational efficiency, and harm-aware ethical impacts.

To measure algorithmic validity and operational efficiency, future pilots should calculate entity resolution precision and recall ratios. Within the context of disaster victim identification, statistical errors carry profound humanitarian consequences. While false positives impose severe psychological trauma on families, false negatives effectively obstruct time-sensitive rescue interventions ( Kreutzer et al., 2025). Systems should also be tested on uncertainty calibration to ensure the AI accurately reflects its confidence levels and its ability to successfully prevent or detect duplicate records. Operational efficiency should be systematically measured using metrics such as time-to-triage, time-to-verification and overall identification latency. For sustainability, these performance indicators should be balanced against the number of cases requiring human escalation, to avoid overloading humanitarian analysts within the system.

Beyond technical performance, the system requires a comprehensive ethical and harm-mitigation evaluation. A primary metric in this assessment is the “false-alert burden,” which ensures that families are protected from the re-traumatizing effects of low-confidence or premature disclosures. To avoid the performative nature of some AI deployments, notification mechanisms must be refined through survivor-led feedback loops that emphasize culturally competent communication ( Elrha, 2025). The architecture’s integrity is maintained through ongoing algorithmic consequence analysis and data minimization audits. Such measures are vital to prevent the dual hazards of unintended surveillance and the systemic redirection of search efforts away from digitally excluded migrant cohorts ( Hoffmann, 2020).

### Failure modes and non-deployment conditions

A critical element of any sociotechnical system in high-stakes crisis environments is the ability to recognize when it should not be used. As this framework manages surveillance-sensitive data and guides potentially life-altering decisions about vulnerable populations, technological progress should never take priority over the fundamental humanitarian imperative to “do no harm” ( Sandvik et al., 2017). The architecture sets clear operational “red lines” to prevent the system from unintentionally replicating systemic violence or surveillance risks. If any of the following failure modes and non-deployment conditions are present, system deployment must be suspended or specific modules disabled:

***Governance and consent deficits***

Lack of Consent: Deployment is not allowed if informed proxy consent from families or legal humanitarian representatives is not obtained, or if the operational environment does not permit the dynamic withdrawal of consent as required in the system’s Cross-layer Controls.

Lack of trusted humanitarian governance: The framework should not be deployed if it cannot be managed or overseen by independent, trusted humanitarian NGOs or multi-stakeholder ethics boards, because it takes the rights-based mandate out of the system ( Metcalfe and Dencik, 2019).

Lack of redress mechanisms: The system cannot function without clear, accessible and independent channels for families and affected communities to report harm, correct false matches, or request data deletion.

***Security and misuse risks***

High risk of law-enforcement misuse: The system should be suspended if changing geopolitical contexts or weak institutional firewalls create an unacceptable risk of surveillance creep ( Latonero & Kift, 2018), where border control or state security agencies might repurpose humanitarian intelligence for deportations, detention or criminalization.

Inability to protect family-provided data: If the host organization does not have the required cybersecurity infrastructure for encrypted, voluntarily provided or highly sensitive data (Tier 3), then the ingestion of such data must be stopped.

***Data and algorithmic integrity failures***

Platform data obtained through ethically questionable means: The system enforces a zero-tolerance policy for digital traces sourced from state-sponsored surveillance or commercial brokers operating in violation of platform privacy. Any data acquisition method that contradicts human rights guidelines is explicitly prohibited.

Unreliable or non-representative data: If the ingested data is compromised by state-sponsored disinformation or digital invisibility, deployment should be paused. Processing skewed data would actively misdirect humanitarian resources and increase representational violence ( Hoffmann, 2020).

Inability to audit AI outputs: High-risk components should not be deployed if their algorithmic outputs remain unauditable by XAI tools or immutable logging. In high-stakes environments, a lack of transparency significantly decreases the ability of affected populations to challenge life-altering algorithmic decisions ( Rudin, 2019).

Insufficient human review capacity: The framework can only work safely if the managing organization has the personnel to maintain the required human-in-the-loop safeguards for reviewing AI-flagged anomalies.

By formalizing these non-deployment conditions, the framework recognizes that in certain volatile or under-resourced contexts, the most ethical humanitarian decision is to refrain from data processing entirely.

## 5. Discussion

This architecture integrates multimodal digital traces with analytical pipelines and embedded ethical safeguards, aligning with studies on digital platforms in crisis response ( W3, 2025). Consistent with studies that used crowdsourced data for earthquake damage assessment or flood detection, the current architecture shows how unconventional data sources such as social media posts, geolocation metadata, and ephemeral online signals, can provide actionable intelligence in contexts of heightened humanitarian risk ( Li et al., 2023; Han et al., 2024).

This work integrates diverse inputs through entity resolution and multimodal fusion. It utilizes probabilistic and machine learning approaches to reconstruct timelines, migration trajectories, and disappearance dynamics, similar to previous initiatives that have integrated diverse records for crisis mapping and forensic investigation ( Xu et al., 2023; Gintova, 2019). The architecture applies transformer models and graph neural networks to reveal risk patterns, consistent with prior research on geospatial and temporal reconstruction ( Mızrak, 2024; Ellouze et al., 2025). The integration of explainable AI techniques and stringent validation approaches addresses current discussions on transparency, accountability, and trust in algorithmic systems utilized in humanitarian operations ( Uluğ et al., 2023; Theja Bhavaraju et al., 2019).

Ethical considerations are fundamental to system design. Prior studies on digital humanitarianism highlighted the risks linked to misinformation, surveillance, and privacy violations in the utilization of crisis data ( Han et al., 2024; Kovras and Robins, 2016). This study incorporates adversarial debiasing, community-driven validation, and rights-based audit mechanisms, thereby aligning with international principles of data responsibility and ethical AI ( Walk et al., 2023; Zhou et al., 2025). Provenance tracking and de-duplication functions effectively tackle operational challenges in humanitarian data integration, as redundant or unverifiable information often compromises efficiency and credibility ( Gintova, 2019).

Despite these improvements, the framework is based on digital traces, which calls for a critical reflection on the quality, bias, and representativeness of data. Migratory populations do not leave equivalent digital traces. While some migrants are equipped with smartphones, stable internet access or supportive digital communities, a substantial portion are “digitally invisible” because of poverty, gender-based restrictions, language barriers, restricted access to platforms, or vulnerability. Dekker et al. (2018) notice that even among asylum seekers who actively utilize social media for navigational purposes, engagement remains shaped and constrained by valid concerns regarding state surveillance.

A fundamental rule of this infrastructure is that the absence of digital data does not denote an absence of humanitarian need. The biases in representativeness and validity have ethical consequences as they may lead to the misallocation of search resources, further marginalizing those who are “digitally invisible” ( Tjaden, 2021). To mitigate systemic biases and amplify blind spots, the framework requires all AI-generated outputs to be triangulated against qualitative ground-truth reports ( Talabi et al., 2022). While entity resolution and spatial modeling offer potential insights, natural biases of digital data necessitate the system be utilized as a supplementary investigative tool rather than an absolute proxy for migratory realities. Furthermore, the sensitive nature of biometric and geolocation data requires continuous strict supervision to mitigate the ethical and privacy concerns that are inherent in post-mortem inquiries. To execute the harm-aware evaluation metrics proposed in this framework, the system is modular and scalable but it requires validation in field deployment. Future research should emphasize pilot implementations in partnership with NGOs, intergovernmental organizations, and impacted communities to assess practicality, ethical precautions, and policy implications in practical contexts.

## 6. Implementation and expected value

As the proposed framework is designed as a scalable and modular sociotechnical infrastructure, it is expected to be implemented through collaborative initiatives between humanitarian informatics researchers, forensic experts, and digital ethnographers. The research community can utilize the novel typology of digital trace types to standardize the collection of decentralized evidence. The proposed modular architecture is designed to be adopted by scholars and humanitarian actors as a scalable and methodologically reproducible framework for the validation of digital traces in migration research. By leveraging interoperable standards, researchers can ensure that their technical artifacts are compatible with international human rights and FAIR data principles.

The framework addresses critical structural deficiencies in current migratory data infrastructures, specifically the “informational blind spots” found in different migration routes. By integrating multimodal traces, the system could facilitate a more robust and fine-grained spatiotemporal reconstruction of migrant trajectories. Unlike traditional border registers that often fail to capture the full scope of disappearances, this method may offer a bridge between computational innovation and rights-based governance. Furthermore, incorporating safeguards such as dynamic consent and explainable AI can advance the principles of “Data Justice.” This may ensure that technical innovation does not undermine participant dignity or privacy.

## 7. Conclusion

This study proposes a conceptual, ethics-integrated framework for transforming fragmented digital traces into unified humanitarian intelligence. The study constitutes a theoretical and operational framework for the balance between computational innovation and robust institutional accountability, through the design of a new typology and a modular architecture. The framework is based on advanced AI and spatiotemporal modeling, but we emphasize its current conceptual status. Prospective applications such as predictive mobility modeling need extensive testing in a sandbox before being considered ready for the field. Future work should emphasize empirical validation via operational pilots. Such studies are essential to evaluate the efficacy of harm-aware metrics in mitigating risks while optimizing triage and identification accuracy in live environments. Therefore, the success of the infrastructure depends not only on its technical capabilities, but also on its consistent commitment to survivor-led governance and data justice.

There are two major limitations. First, evolving platform features, or content norms and access regimes require longitudinal benchmarking and cross-platform replication. Second, the lack of verifiable ground truth data makes validation difficult. This requires centralized partnerships with NGOs and forensic authorities for verified labels and survivor-centred feedback. Future directions include piloting domain-specific multilingual models, co-designing evaluation metrics that balance precision/recall with harm-aware metrics (e.g., false-alert burden on families), and prospective trials to measure impacts on triage time, identification rates and notification quality.

## Ethics and consent

Ethical approval and consent were not required.

## Data Availability

No data is associated with this article.
