# Supplementary material for: Humanitarian data infrastructures for missing migrants: A multimodal and ethics-integrated framework
Source: Open Res Eur. 2026 Jun 17;6:118. Originally published 2026 Apr 16. [Version 2] doi: 10.12688/openreseurope.23124.2 (PMC13332383; doi:10.12688/openreseurope.23124.2)
Supplement: Peer review history

# Reviewer report: Reviewer response for version 2

**DOI:** 10.21956/openreseurope.26194.r76051

**Published:** 2026-07-03

**Authors:** Tutku Tuncalı Yaman

**Affiliations:** 1 Department of Management Information Sciences, Marmara University, Istanbul, Turkey

This manuscript presents an integrated conceptual framework for humanitarian data infrastructures concerning missing migrants. It combines a typology of digital traces, modular data-processing architecture, evidence integration, AI-supported analysis, governance safeguards, and rights-based ethical controls. The topic is important, timely, and inherently interdisciplinary.

 In the earlier version, the manuscript’s central contribution, technical workflow, trace typology derivation, implementation boundaries, ethical safeguards, reproducibility, and performance claims required substantial strengthening. The revised version addresses these concerns thoroughly and constructively.

 The most important revisions include:

- A clearer definition of the manuscript’s central methodological contribution as an integrated framework rather than an unspecified combination of typology, architecture, and policy vision.
- A transparent hybrid derivation process for the digital trace typology.
- A trace-risk matrix that distinguishes evidentiary value, ethical sensitivity, consent requirements, reliability, accessibility, potential harms, and suitability for automated processing.
- A structured classification of data sources and access conditions, including public/open data, API-accessible data, voluntarily submitted data, NGO/institutional records, sensitive restricted data, and data not recommended for routine collection.
- A more operational governance model addressing proxy agency, consent withdrawal, deletion pathways, role-based access control, retention limits, auditability, redress, and safeguards against surveillance or deportation-related repurposing.
- A reproducible workflow that separates minimum requirements, optional analytical extensions, high-risk components, and future research components.
- A synthetic pilot scenario that demonstrates how conflicting multimodal evidence may be processed while preserving human oversight and ethical controls.
- A harm-aware validation framework that distinguishes algorithmic validity, operational efficiency, and impacts on affected individuals and communities.
- A stronger treatment of digital invisibility, unequal access, representativeness, bias, and the principle that absence of digital traces must not be interpreted as absence of risk, need, or movement.
- Explicit failure modes and non-deployment conditions that appropriately prioritize protection from harm over technical expansion.

The revised manuscript is now more rigorous, more transparent, and more appropriately cautious in its claims. It should be read as a conceptual and methodological contribution that requires subsequent sandbox validation, participatory review, and field-based assessment before operational deployment. Within that scope, the article makes a useful contribution to humanitarian informatics, migration-data research, responsible AI, and rights-based data-governance scholarship.

Are sufficient details provided to allow replication of the method development and its use by others?

No

Is the description of the method technically sound?

Partly

Are the conclusions about the method and its performance adequately supported by the findings presented in the article?

Partly

Is the rationale for developing the new method (or application) clearly explained

Partly

If any results are presented, are all the source data underlying the results available to ensure full reproducibility?

No source data required

Reviewer Expertise:

My areas of expertise include Management Information Systems, artificial intelligence and explainable AI, data analysis, data governance, digital data infrastructures, sociotechnical information systems, research methodology, and the application of computational methods in interdisciplinary domains. I am particularly able to assess the article’s methodological design, technical architecture, data governance and interoperability claims, ethical safeguards, reproducibility, and implementation feasibility. I also have research experience with multimodal, sensitive, and context-dependent data infrastructures.

I confirm that I have read this submission and believe that I have an appropriate level of expertise to confirm that it is of an acceptable scientific standard.

# Reviewer report: Reviewer response for version 1

**DOI:** 10.21956/openreseurope.25016.r73589

**Published:** 2026-05-07

**Authors:** Nur Kuban Torun

**Affiliations:** 1 Management Information Systems, Bilecik Sheikh Edebali University, Bilecik, Turkey

One of the biggest problems of our time is non-structural migration. Tracking those forced to migrate, especially due to factors like war, increases the workload for countries. In this context, the subject matter and the approaches presented in this research have the potential to significantly contribute to tracking migrants through digital platforms. In light of all this, the study is a suitable and effective contribution to the journal's content.

Are sufficient details provided to allow replication of the method development and its use by others?

Yes

Is the description of the method technically sound?

Yes

Are the conclusions about the method and its performance adequately supported by the findings presented in the article?

Yes

Is the rationale for developing the new method (or application) clearly explained

Yes

If any results are presented, are all the source data underlying the results available to ensure full reproducibility?

Yes

Reviewer Expertise:

data mining, artifical intellegent, digital transformation

I confirm that I have read this submission and believe that I have an appropriate level of expertise to confirm that it is of an acceptable scientific standard.

# Reviewer report: Reviewer response for version 1

**DOI:** 10.21956/openreseurope.25016.r72555

**Published:** 2026-05-04

**Authors:** Tutku Tuncalı Yaman

**Affiliations:** 1 Department of Management Information Sciences, Marmara University, Istanbul, Turkey

**Summary of the article**

 This article proposes a multimodal and ethics-integrated humanitarian data infrastructure for missing migrants. The authors argue that existing data systems, including border registers, forensic databases, missing-person registries, and humanitarian reporting systems, are fragmented and often unable to capture the full scope of migrant disappearances. To address this problem, the article introduces a typology of 32 digital trace types across ten domains and proposes a modular architecture composed of ingestion and verification, data processing, evidence integration, AI processing, analysis/intelligence, and cross-layer ethical controls. The framework aims to integrate social media traces, geospatial markers, multilingual content, media evidence, behavioral indicators, and psychosocial signals into a responsible data infrastructure. It also emphasizes FAIR principles, explainable AI, provenance tracking, dynamic consent, auditability, human-in-the-loop governance, and data justice.

 The article is timely and addresses a highly important humanitarian problem. Its main strengths are its broad recognition of multimodal digital traces, its attempt to embed ethics across the data lifecycle, and its effort to connect sociotechnical systems thinking with humanitarian data infrastructure. However, the manuscript requires major revision before it can be considered scientifically sound as a method article. The central problems concern technical under-specification, lack of replicability, insufficient justification of the typology, overstatement of system capabilities, and inadequate operationalization of ethical safeguards.

 Major points that must be addressed to make the article scientifically sound

**1. Clarify the exact methodological contribution**

 The manuscript currently combines a typology, a conceptual architecture, an AI pipeline, a governance framework, and a policy-facing humanitarian system. These are all valuable, but the central contribution is not sufficiently focused.

 The authors should state explicitly whether the article’s primary contribution is:

 1.    a typology of digital traces for missing-migrant research; 

 2.    a modular humanitarian data infrastructure; 

 3.    an ethics-integrated governance model; 

 4.    a technical AI pipeline; 

 5.    or an integrated conceptual framework combining all of these. 

 This clarification is necessary because the standards of evaluation differ for each contribution. A typology requires transparent derivation and validation. A technical architecture requires implementation specifications. A method article requires sufficient detail for replication. A governance model requires explicit accountability, consent, access, and risk-management procedures.

**2. Provide a transparent derivation process for the typology**

 The typology of 32 digital trace types is one of the most promising parts of the manuscript. However, the manuscript does not explain how these trace types were identified or classified. The authors should add a subsection explaining the development of the typology.

 This section should specify:

 1.    the literature base used to identify trace types; 

 2.    the selection criteria; 

 3.    whether the process was deductive, inductive, or hybrid; 

 4.    how overlapping categories were resolved; 

 5.    whether domain experts reviewed the typology; 

 6.    whether the typology was checked against existing humanitarian or forensic workflows. 

 The authors should also add a trace-risk matrix. Each trace type should be evaluated according to evidentiary value, sensitivity, consent requirements, reliability, platform accessibility, risk of harm, and suitability for automated processing. This is particularly important because the current typology places very different data types side by side, such as public hashtags, private read receipts, facial recognition tags, voice notes, memes, geotags, DNA/forensic records, and family testimony. These traces differ fundamentally in evidentiary status and ethical risk.

**3. Distinguish public, consent-based, restricted, and non-recommended data sources**

 The manuscript implies that social media traces and platform-specific signals can be routinely collected and analyzed. This is not always legally, ethically, or technically realistic. Many of the proposed trace types, such as “last seen online,” typing indicators, read receipts, private voice notes, direct messages, account deactivation, algorithmic boosting, and cross-platform interaction histories, may not be publicly available and may require explicit consent or legal authority.

 The authors should classify all data sources into categories such as:

 1.    public/open data; 

 2.    voluntarily submitted family/community data; 

 3.    NGO or institutional case records; 

 4.    platform API-accessible data; 

 5.    sensitive restricted data; 

 6.    data not recommended for routine collection. 

 This classification is necessary for technical feasibility and ethical legitimacy. The current manuscript does not sufficiently separate feasible public digital trace analysis from highly sensitive or legally restricted data processing.

**4. Operationalize the ethical governance model**

 The manuscript repeatedly refers to dynamic consent, data justice, auditability, privacy by design, survivor-led validation, and human-in-the-loop governance. These principles are appropriate but remain too general. Given the vulnerability of missing migrants and their families, the manuscript must specify how these principles would operate in practice.

 The authors should address:

 1.    how consent is obtained when the data subject is missing, deceased, detained, undocumented, or unreachable; 

 2.    who may provide consent on behalf of a missing person; 

 3.    how consent can be withdrawn; 

 4.    how family-submitted data are protected from misuse; 

 5.    how cross-border data transfer is handled; 

 6.    how law-enforcement or border-control repurposing is prevented; 

 7.    how access rights are tiered across NGOs, forensic experts, researchers, families, and public authorities; 

 8.    how long data are retained; 

 9.    how audit logs are maintained; 

 10.    what redress mechanisms exist in case of error or harm. 

 The humanitarian AI ethics literature shows that risks related to autonomy, non-maleficence, beneficence, and justice become especially acute when personal data and AI are used in crisis contexts. Kreutzer et al.’s scoping review should be used to strengthen this section. 

**5. Convert the architecture from a technology catalogue into a reproducible workflow**

 The article currently lists a large number of technologies and standards, but it does not integrate them into a sufficiently clear workflow. The authors should separate the architecture into:

 1.    required minimum components; 

 2.    optional analytical extensions; 

 3.    high-risk components requiring special approval; 

 4.    future research components. 

 For each module, the authors should specify inputs, outputs, standards, decision rules, validation checks, and responsible actors. An operational table would substantially improve the technical soundness and replicability of the article.

**6. Add a worked example or pilot scenario**

 The manuscript would be much stronger if it included a worked example. This does not require real sensitive data. A fictional or synthetic case could demonstrate the workflow without ethical risk.

 For example, the authors could present a hypothetical case involving:

 1.    a family report of a missing person; 

 2.    a public social media appeal; 

 3.    a geotagged post; 

 4.    a witness comment; 

 5.    a visual image; 

 6.    conflicting location information; 

 7.    human review; 

 8.    case confidence scoring; 

 9.    generation of a humanitarian action report. 

 This would show how the method functions and would make the architecture more comprehensible and testable.

**7. Define validation and evaluation metrics**

 The article claims that the framework can support identification, early warning, risk scoring, policy intelligence, and family notification. These claims require a validation plan. The authors should define metrics for future evaluation, even if no pilot has yet been conducted.

 Potential metrics include:

 1.    entity resolution precision and recall; 

 2.    false positive and false negative rates; 

 3.    time to triage; 

 4.    time to verification; 

 5.    identification latency; 

 6.    proportion of cases requiring human escalation; 

 7.    uncertainty calibration; 

 8.    false-alert burden on families; 

 9.    data minimization compliance; 

 10.    number of prevented or detected duplicate records; 

 11.    family notification quality; 

 12.    harm-aware evaluation metrics. 

 This point is crucial. Explainable AI tools such as SHAP and LIME do not by themselves demonstrate validity, fairness, or safety. The authors should specify what performance means in this humanitarian context and how performance will be evaluated without increasing harm.

**8. Reduce overclaims about system performance**

 Several claims in the manuscript imply that the system can deliver operational intelligence, support early warning, enable family notification, and produce policy accountability. Since the article does not present implementation or evaluation results, these claims should be softened.

 The authors should replace phrases such as:

 •    “the system enables” 

 •    “the framework ensures” 

 •    “the architecture guarantees” 

 •    “the system delivers actionable intelligence” 

 with more cautious formulations:

 •    “the framework is designed to support” 

 •    “the architecture could facilitate” 

 •    “the proposed model aims to provide” 

 •    “future pilots should assess whether the framework can improve…” 

 This revision would make the conclusions more proportionate to the evidence presented.

**9. Strengthen discussion of data quality, bias, and representativeness**

 The article should discuss the limitations of social media and digital traces more critically. Digital traces are not evenly distributed. Some migrants may have smartphones, stable internet access, public social media presence, and supportive digital communities; others may be digitally invisible due to poverty, fear of surveillance, gendered restrictions, language barriers, platform access, or legal vulnerability.

 The absence of digital trace data should not be interpreted as absence of need, absence of risk, or absence of movement. Digital migration data studies have repeatedly emphasized issues of representativeness, validity, access, and ethical risk. Tjaden’s review is especially relevant here. Dekker et al. also show that social media are important for asylum migrants but are shaped by uneven access and surveillance concerns.

**10. Add a section on failure modes and non-deployment conditions**

 The manuscript should explicitly discuss when the framework should not be deployed. This is essential because the framework concerns vulnerable populations, surveillance-sensitive data, and potentially life-altering decisions.

 The authors should add a section titled “Failure modes and non-deployment conditions.” This section could include:

 1.    insufficient consent; 

 2.    high risk of law-enforcement misuse; 

 3.    inability to protect family-provided data; 

 4.    lack of trusted humanitarian governance; 

 5.    insufficient human review capacity; 

 6.    unreliable or non-representative data; 

 7.    inability to audit AI outputs; 

 8.    absence of redress mechanisms; 

 9.    platform data obtained through ethically questionable means. 

 Such a section would show that the authors understand the risks of humanitarian data infrastructures and are not simply promoting technological expansion.

**Minor comments**

 1.    The article should define “digital traces,” “ambient traces,” “actionable intelligence,” “data justice,” “survivor-led governance,” and “humanitarian data infrastructure” more precisely. 

 2.    The five research questions are too broad. They should be consolidated or aligned more directly with the article’s method contribution. 

 3.    Table 2 is useful but visually dense. It should be redesigned to improve readability. 

 4.    Figure 1 contains many components but does not clearly communicate data flow, governance layers, or decision points. 

 5.    The manuscript should distinguish between forensic, humanitarian, research, and policy uses of the system. 

 6.    Some references appear only loosely connected to the claims they support. The authors should check that each citation directly supports the statement for which it is used. 

 7.    The language should be revised for clarity and precision. Some claims are too general, and some technical expressions require explanation. 

 8.    The article should avoid presenting speculative AI applications as if they are ready for operational deployment. 

 9.    The conclusion should be rewritten to reflect the conceptual status of the framework. 

 10.    A supplementary appendix mapping trace types to sources, standards, risks, and validation needs would significantly improve the manuscript. 

**Overall recommendation**

 Overall, the manuscript addresses a significant and timely humanitarian problem and proposes a potentially valuable framework. However, the article is not yet sufficiently developed as a method article. The main contribution needs to be clarified, the typology requires a transparent derivation process, and the architecture should be converted from a high-level conceptual model into a reproducible methodological workflow. The ethical safeguards also need to be operationalized in much greater detail, particularly given the sensitivity of biometric, geolocation, social media, family-submitted, and potentially forensic data.

**The following points must be addressed to make the article scientifically sound:**

1. Clearly define the primary methodological contribution of the article.
2. Explain how the 32 digital trace types were identified, selected, categorized, and validated.
3. Add a trace-risk matrix evaluating each trace type in terms of sensitivity, evidentiary value, reliability, consent requirements, access conditions, and potential harm.
4. Distinguish public, consent-based, restricted, and non-recommended data sources.
5. Provide a reproducible workflow with inputs, outputs, metadata requirements, validation rules, human-review triggers, and uncertainty handling for each module.
6. Operationalize the ethical governance model, including consent, withdrawal, access control, retention, deletion, cross-border transfer, audit, redress, and misuse-prevention mechanisms.
7. Add a worked example or hypothetical case showing how data move through the proposed system.
8. Define validation and evaluation metrics, including entity-resolution accuracy, false-alert burden, triage time, uncertainty calibration, identification latency, and harm-aware performance indicators.
9. Reduce unsupported claims about system performance and distinguish clearly between proposed, implemented, and future capabilities.
10. Expand the discussion of data quality, representativeness, platform dependency, digital invisibility, and non-deployment conditions.

With these revisions, the manuscript could make a useful contribution to humanitarian informatics, digital migration studies, and ethics-integrated data infrastructure design.

Are sufficient details provided to allow replication of the method development and its use by others?

No

Is the description of the method technically sound?

Partly

Are the conclusions about the method and its performance adequately supported by the findings presented in the article?

Partly

Is the rationale for developing the new method (or application) clearly explained

Partly

If any results are presented, are all the source data underlying the results available to ensure full reproducibility?

No source data required

Reviewer Expertise:

My areas of expertise include Management Information Systems, artificial intelligence and explainable AI, data analysis, data governance, digital data infrastructures, sociotechnical information systems, research methodology, and the application of computational methods in interdisciplinary domains. I am particularly able to assess the article’s methodological design, technical architecture, data governance and interoperability claims, ethical safeguards, reproducibility, and implementation feasibility. I also have research experience with multimodal, sensitive, and context-dependent data infrastructures.

I confirm that I have read this submission and believe that I have an appropriate level of expertise to confirm that it is of an acceptable scientific standard, however I have significant reservations, as outlined above.

## References

[1] : Measuring migration 2.0: a review of digital data sources. *Comparative Migration Studies* .2021;9(1) : 10.1186/s40878-021-00273-x 10.1186/s40878-021-00273-x

[2] : Ethical implications related to processing of personal data and artificial intelligence in humanitarian crises: a scoping review. *BMC Medical Ethics* .2025;26(1) : 10.1186/s12910-025-01189-2 10.1186/s12910-025-01189-2 40229745 PMC11998222

[3] : Nowcasting Daily Population Displacement in Ukraine through Social Media Advertising Data. *Population and Development Review* .2023;49(2) : 10.1111/padr.12558 231-254 10.1111/padr.12558

[4] : Smart Refugees: How Syrian Asylum Migrants Use Social Media Information in Migration Decision-Making. *Social Media + Society* .2018;4(1) : 10.1177/2056305118764439 10.1177/2056305118764439

[5] : What is data justice? The case for connecting digital rights and freedoms globally. *Big Data & Society* .2017;4(2) : 10.1177/2053951717736335 10.1177/2053951717736335

[6] : Technocolonialism: Digital Innovation and Data Practices in the Humanitarian Response to Refugee Crises. *Social Media + Society* .2019;5(3) : 10.1177/2056305119863146 10.1177/2056305119863146

# Response

**Published:** 2026-06-01

**Authors:** Fevzi Esen

**Affiliations:** Health Informatics, Saglik Bilimleri Universitesi, Istanbul, İstanbul, Turkey

**Dear Editor and Reviewers,**

 We would like to express our gratitude for your constructive, and comprehensive review of our manuscript titled “Humanitarian data infrastructures for missing migrants: A multimodal and ethics-integrated framework”. Your feedback has provided an invaluable blueprint for elevating this work from a high-level conceptual model into a robust, operationally sound, and replication-ready method article. We have addressed major and minor comment in detail. The manuscript has been structurally expanded to include transparent derivation processes, an active trace-risk matrix, an operational workflow categorization, a complete synthetic pilot scenario, a harm-aware validation plan, and clear boundaries for non-deployment. Furthermore, all terminology has been sharpened, and deterministic claims have been systematically replaced with design-oriented language. Below is our comprehensive, point-by-point response detailing the specific revisions made to the text. All text modifications, updates, and newly added sections in the revised manuscript have been highlighted in red font.  

**Part 1: Response to Major Comments**  

**1. Clarify the exact methodological contribution** **Reviewer Comment:** The manuscript currently combines a typology, a conceptual architecture, an AI pipeline, a governance framework, and a policy-facing humanitarian system. These are all valuable, but the central contribution is not sufficiently focused. The authors should state explicitly whether the article’s primary contribution is a typology, an infrastructure, a governance model, a pipeline, or an integrated framework.

**Response:** We appreciate the need for clear categorization. The primary methodological contribution of this paper is an unified, integrated conceptual framework that combines all of these sub-components. Rather than treating data categorization, technical processing, and ethical governance as separate issues, this article argues that a rights-based response throughout the migration cycle requires their structural convergence. To make this explicit, we have rewritten the final paragraphs of the Introduction to clarify the integrated analytical approach of our methodological framework and guide the reader on how each sub-component supports replication.

**2. Transparent derivation process for the typology and Trace-Risk Matrix** **Reviewer Comment:** The manuscript does not explain how these trace types were identified or classified. The authors should add a subsection explaining the development of the typology (literature base, selection criteria, hybrid process, expert review, forensic workflows). The authors should also add a trace-risk matrix evaluating each trace type.

**Response:** We have expanded the text to provide full methodological transparency regarding how the 32 digital trace types were derived and verified. We added a text block explaining the hybrid derivation process, noting that a deductive approach extracted established data categories from crisis informatics literature, while an inductive approach captured emerging ambient signals. We outline our specific inclusion criteria and detail how the typology was reviewed by international working groups and validated against existing humanitarian workflows like the ICRC Trace the Face initiative and Interpol DVI protocols. Furthermore, to address the core of the reviewer's concern, we have built an original Table 3 (Trace-Risk Matrix for Missing Migrant Digital Evidence). This matrix explicitly evaluates public, visual, ambient, psychosocial, and institutional trace categories across seven precise metrics: evidentiary value, ethical sensitivity, consent requirements, reliability, platform accessibility, risk of harm, and automated AI processing suitability.

**3. Distinguish public, consent-based, restricted, and non-recommended data sources** **Reviewer Comment:** The manuscript implies that social media traces can be routinely collected and analyzed. This is not realistic. The authors should classify all data sources into categories (public, voluntarily submitted, NGO records, API-accessible, restricted, non-recommended) for technical feasibility and ethical legitimacy.

**Response:** We completely agree that technical extractability does not equate to ethical legitimacy. To address this boundary, we added a dedicated subsection titled “Data Accessibility and Ethical Collection Tiers”. We structurally segregate all 32 trace types into six distinct operational levels:

1. Public/Open Data (e.g., public hashtags and alerts)
2. Platform API-Accessible Data (e.g., aggregated trends or topologies via endpoints)
3. Voluntarily Submitted Family/Community Data (operationalized strictly via privacy-preserving “data donation” methodologies)
4. NGO or Institutional Case Records (governed by formal data-sharing agreements and purpose limitations)
5. Sensitive Restricted Data (highly regulated forensics, limited to authorized entities)
6. Data Not Recommended for Routine Collection (e.g., passive cross-platform interaction histories and scraping tracking metrics without consent, which carry risks that exceed humanitarian benefits).

**4. Operationalize the ethical governance model** **Reviewer Comment:** The manuscript repeatedly refers to dynamic consent, data justice, etc., but these remain too general. Specify how these principles would operate in practice (missing subjects, proxy agency, withdrawal, cross-border transfers, firewalls, retention, and redress). Kreutzer et al.’s scoping review should be used to strengthen this section.

**Response:** We have deeply refined and operationalized our governance mechanisms to replace high-level principles with concrete procedural safeguards. We restructured Section 4 under “Operationalizing Ethical Governance and Rights-Based Safeguards” using Kreutzer et al. (2025) as our theoretical anchor to address the acute risks of crisis-context data processing. The framework now explicitly defines:

- Proxy Agency: A delegated model where verified next-of-kin or humanitarian representatives manage consent for unreachable subjects.
- Consent Withdrawal: A secure pathway triggering a “deletion cascade” across active processing pipelines.
- Firewalls & Access Tiering: Strict purpose limitations and Role-Based Access Control (RBAC) that create a legal and technical firewall blocking state security or law enforcement from repurposing data for deportations.
- Lifecycle & Auditability: Automated, tamper-evident audit logs using W3C PROV standards and defined retention schedules where ephemeral traces are purged after triage.
- Redress Mechanisms: Independent ethics boards and Data Protection Officers (DPOs) authorized to suspend system modules in case of false matches or harm.

**5. Convert the architecture into a reproducible workflow** **Reviewer Comment:** The article currently lists a large number of technologies but does not integrate them into a clear workflow. Separate the architecture into: required minimum, optional extensions, high-risk components, and future research components. An operational table is needed.

**Response:** To transition the manuscript from a “technology catalogue” to a highly reproducible, structured methodology, we added a comprehensive text block and built a rigorous operational matrix: Table 6 (Operational Workflow and Component Categorization). This table stratifies the 5 system cores across the four requested operational tiers:

1. Required Minimum Components: Base ingestion, deduplication, and triage systems.
2. Optional Analytical Extensions: Advanced computational NLP and thematic clustering modules.
3. High-Risk Components Requiring Special Approval: Automated entity resolution and modules processing Tier 5 biometrics, requiring explicit legal mandates, dual-authorization, and explainable AI (XAI) audits.
4. Future Research Components: Experimental predictive mobility modeling and synthetic narrative stress-testing, siloed as advisory.

For each tier, the table clearly specifies exact inputs, outputs, governing standards (e.g., ISO/IEC 27001, W3C PROV, FAIR), exemplary decision rules, validation checks, and responsible human actors in the loop.

**6. Add a worked example or pilot scenario** **Reviewer Comment:** Include a fictional or synthetic case study to demonstrate the workflow without ethical risk, tracking a family report, a social media appeal, a geotagged post, a witness comment, a visual image, conflicting location data, human review, scoring, and an action report.

**Response:** We have acted on this recommendation by creating an exhaustive walk-through scenario designed to explicitly test our framework's components. The scenario maps directly to the active system modules defined in our paper. The narrative follows the processing of a synthetic case (“Subject M”) across five distinct operational phases:

- Phase 1 (Ingestion): Translates a family intake file and visual image into a public social media appeal with W3C PROV tracking.
- Phase 2 (Processing/AI Pipeline): Runs CLIP-based Image-Text Retrieval on a scraped, geotagged post to find an 88% biometric match, while parsing a text-based witness comment.
- Phase 3 (Integration): Detects conflicting location information between the metadata geotag (coastal hub) and the parsed comment text (inland route).
- Phase 4 (Controls): Suspends automated processing and triggers an intensive human review by a digital ethnographer utilizing an Explanation Generator (LIME/SHAP) to identify text-based misinformation patterns.
- Phase 5 (Analysis): Calculates a case confidence scoring (82%), respects high-risk notification thresholds, and successfully outputs an OCHA-compliant humanitarian action report for field rescue teams.

**7. Define validation and evaluation metrics** **Reviewer Comment:** Define metrics for future evaluation. Explainable AI tools such as SHAP and LIME do not by themselves demonstrate validity, fairness, or safety. Specify what performance means in this humanitarian context and how performance will be evaluated without increasing harm.

**Response:** We completely validate this philosophical position: model interpretability via SHAP/LIME does not automatically translate to operational safety or fairness. To establish clear benchmarks for evaluation, we added two comprehensive paragraphs outlining a harm-aware validation plan that defines “performance” through the lens of dignity and harm reduction. The text explicitly integrates all 12 evaluation metrics requested by the reviewer, organizing them logically into three fields:

- Algorithmic Validity: Tracking entity resolution precision and recall, false positive and false negative rates (noting that false positives inflict immense trauma on families), uncertainty calibration, and the tracking of prevented/detected duplicate records.
- Operational Efficiency: Benchmarking time to triage, time to verification, identification latency, and the proportion of cases requiring human escalation to verify organizational sustainability.
- Harm-Aware Impact: Measuring the false-alert burden on families, family notification quality (via survivor-led qualitative feedback loops), data minimization compliance audits, and broader systemic harm-aware evaluation metrics to ensure digitally invisible or marginalized groups are not structurally neglected.

**8. Reduce overclaims about system performance** **Reviewer Comment:** Several claims in the manuscript imply that the system can deliver operational intelligence, support early warning, enable family notification, and produce policy accountability. Since the article does not present implementation or evaluation results, these claims should be softened. Replace phrases like “the system enables,” “the framework ensures,” etc.

**Response:** We acknowledge this necessary correction. Because our proposed model has not yet undergone field testing, deterministic verbs have been replaced with design-oriented, probabilistic, and humble language across all sections, including the Abstract, Introduction, Results, Expected Value, and Conclusion. Key linguistic transitions executed include:

- “the system enables”, “the system is designed to support”
- “the framework ensures”, “the framework seeks to facilitate”
- “the architecture guarantees”, “the architecture is designed to verify” / “aims to ensure”
- “deliver early warnings”, “aim to provide early warnings”
- “by using anti-oppressive design principles, the system guarantees...”, “...the system can promote transparency...”

**9. Strengthen discussion of data quality, bias, and representativeness** **Reviewer Comment:** Discuss the limitations of social media and digital traces more critically. Digital traces are not evenly distributed; some migrants are digitally invisible. The absence of data must not be interpreted as absence of need, risk, or movement. Cite Tjaden and Dekker et al. **Response:** We have fully revised our Discussion section to provide a critique of digital trace dependency. We explicitly address how poverty, language barriers, gendered restrictions, and acute legal vulnerabilities construct “digital invisibility” among migrant cohorts. Citing the critical insights of Dekker et al. (2018), we highlight how smartphone and platform usage is actively constrained by legitimate fears of state surveillance. Most importantly, we have written into our framework the fundamental operational rule that the absence of digital trace data must never be interpreted as an absence of need, an absence of risk, or an absence of movement. We utilize Tjaden (2021) to emphasize that uncritical reliance on digital footprints creates massive ethical risks by potentially misdirecting humanitarian rescue resources away from marginalized, digitally invisible groups.

**10. Add a section on failure modes and non-deployment conditions** **Reviewer Comment:** The manuscript should explicitly discuss when the framework should not be deployed. Add a section titled “Failure modes and non-deployment conditions” including all 9 specified operational deficits.

**Response:** We have built a dedicated new section addressing this mandate, formalizing explicit ethical “red lines” where system operation must be aborted or completely deactivated to prioritize participant safety over software expansion. The new section explicitly maps and operationally defines all 9 conditions requested:

- Governance/Consent Deficits: Halting deployment under insufficient consent states, a lack of trusted humanitarian governance (citing Metcalfe and Dencik, 2019), or an absence of redress mechanisms.
- Security/Misuse Risks: Mandating non-deployment under a high risk of law-enforcement misuse (preventing surveillance creep into border control deportations, citing Latonero and Kift, 2018) or an technical inability to protect family-provided data.
- Data/Algorithmic Integrity Failures: Rejecting processing if data is obtained through ethically questionable means (spyware or illicit brokers), if data is highly unreliable or non-representative, if there is an inability to audit AI outputs due to black-box models (citing Rudin, 2019), or if there is insufficient human review capacity to maintain mandatory human-in-the-loop (HITL) safeguards.

**Part 2: Response to Minor Comments**  

**1. Precise Definitions of Core Terms** **Minor Comment 1:** Precisely define “digital traces,” “ambient traces,” “actionable intelligence,” “data justice,” “survivor-led governance,” and “humanitarian data infrastructure.”

**Response:** We have integrated an explicit definitional paragraph into our Introduction that structurally defines all six terms, providing clear conceptual and epistemological boundaries for our framework.

**2. Tightening Research Questions** **Minor Comment 2:** The five research questions are too broad. Consolidate or align them with the method contribution.

**Response:** We have consolidated the five broad introductory queries into three tightly focused, method-driven research questions that map directly to our central architectural, typological, and rights-based safeguard contributions.

**3. Redesigning Table 2** **Minor Comment 3:** Table 2 is useful but visually dense. Redesign to improve readability.

**Response:** We have completely abandoned the horizontal, split-column presentation of Table 2. It has been entirely redesigned into a clean, vertical, 4-column layout (Trace Category, Specific Data Types, Humanitarian & Analytical Utility, and Methodological Notes) that drastically enhances data legibility and scannability.

**4. Clarification of Data Flow and Figure 1** **Minor Comment 4:** Figure 1 does not clearly communicate data flow, governance layers, or decision points.

**Response:** We have adjusted our descriptive text immediately surrounding Figure 1 to explicitly outline the horizontal pipeline flow. We have further defined how the Cross-layer Controls function as a continuous, foundational baseline that underpins every technical decision node.

**5. Categorization of System User End-Cases** **Minor Comment 5:** Distinguish between forensic, humanitarian, research, and policy uses of the system.

**Response:** We added an introductory paragraph to the operational outputs section that cleanly separates system capabilities based on the explicit end-user: real-time rescue for humanitarian workflows; post-mortem disaster victim identification (DVI) under strict forensic protocols; fully anonymized data pipelines for research digital ethnography; and macro-level trends for policy accountability dashboards.

**6. Reference Audit and Speculative AI Classifications** **Minor Comments 6, 7, and 8:** Check citations directly support claims; revise language for precision; avoid presenting speculative AI applications as ready for operational deployment.

**Response:** We have verified all citations across our bibliography. To ensure speculative or experimental AI tools are not presented as field-ready, our newly implemented Reproducible Workflow (Table 6) expressly isolates unvalidated tools such as predictive mobility modeling and synthetic narrative simulations into Tier 4: Future Research Components, establishing that they must be watermarked and completely decoupled from active field rescue operations. **7. Conceptual Reframing of the Conclusion** **Minor Comment 9:** Rewrite the conclusion to reflect the conceptual status of the framework.

**Response:** The opening paragraph of our Conclusion has been entirely rewritten to underscore the conceptual status of the proposed infrastructure, stating clearly that sandbox validation and field testing are strictly required before operational field readiness can be claimed.

**8. Supplementary Mapping Appendix** **Minor Comment 10:** Add a supplementary appendix mapping trace types to sources, standards, risks, and validation needs.

**Response:** We have generated a comprehensive Supplementary Appendix Table at the very end of the manuscript. This matrix cross-maps each primary trace category against its Primary Source, Legal/Technical Standard (e.g., GDPR, W3C PROV, Interpol DVI, ISO/IEC 27001), Primary Ethical Risk, and corresponding Validation Requirement. We believe these extensive revisions have completely resolved the structural deficiencies noted by the reviewers, turning this manuscript into a field-ready contribution to humanitarian informatics. Sincerely,
